# Conversational Physical Activity Coaches for Spanish and English Speaking Women: A User Design Study

**DOI:** 10.3389/fdgth.2021.747153

**Published:** 2021-10-08

**Authors:** Caroline A. Figueroa, Tiffany C. Luo, Andrea Jacobo, Alan Munoz, Minx Manuel, David Chan, John Canny, Adrian Aguilera

**Affiliations:** ^1^School of Social Welfare, University of California, Berkeley, Berkeley, CA, United States; ^2^School of Public Health, University of California, Berkeley, Berkeley, CA, United States; ^3^Department of Electrical Engineering and Computer Sciences, University of California, Berkeley, Berkeley, CA, United States; ^4^Department of Psychiatry and Behavioral Sciences, Zuckerberg San Francisco General Hospital, University of California, San Francisco, San Francisco, CA, United States

**Keywords:** women, mHealth, user-centered design, low-income, digital divide, chatbots, conversational agents, exercise

## Abstract

**Introduction:** Digital technologies, including text messaging and mobile phone apps, can be leveraged to increase people's physical activity and manage health. Chatbots, powered by artificial intelligence, can automatically interact with individuals through natural conversation. They may be more engaging than one-way messaging interventions. To our knowledge, physical activity chatbots have not been developed with low-income participants, nor in Spanish—the second most dominant language in the U.S. We recommend best practices for physical activity chatbots in English and Spanish for low-income women.

**Methods:** We designed a prototype physical activity text-message based conversational agent based on various psychotherapeutic techniques. We recruited participants through SNAP-Ed (Supplemental Nutrition Assistance Program Education) in California (Alameda County) and Tennessee (Shelby County). We conducted qualitative interviews with participants during testing of our prototype chatbot, held a Wizard of Oz study, and facilitated a co-design workshop in Spanish with a subset of our participants.

**Results:** We included 10 Spanish- and 8 English-speaking women between 27 and 41 years old. The majority was Hispanic/Latina (*n* = 14), 2 were White and 2 were Black/African American. More than half were monolingual Spanish speakers, and the majority was born outside the US (>50% in Mexico). Most participants were unfamiliar with chatbots and were initially skeptical. After testing our prototype, most users felt positively about health chatbots. They desired a personalized chatbot that addresses their concerns about privacy, and stressed the need for a comprehensive system to also aid with nutrition, health information, stress, and involve family members. Differences between English and monolingual Spanish speakers were found mostly in exercise app use, digital literacy, and the wish for family inclusion.

**Conclusion:** Low-income Spanish- and English-speaking women are interested in using chatbots to improve their physical activity and other health related aspects. Researchers developing health chatbots for this population should focus on issues of digital literacy, app familiarity, linguistic and cultural issues, privacy concerns, and personalization. Designing and testing this intervention for and with this group using co-creation techniques and involving community partners will increase the probability that it will ultimately be effective.

## Introduction

Insufficient physical activity is one of the leading risk factors of death worldwide (1). Marginalized groups, such as people of lower socioeconomic status (2), women (3), and ethnic/racial minorities including Latinas (4), are particularly inactive. We need to develop interventions that help marginalized populations increase and maintain healthy physical activity behaviors.

In recent years, digital behavioral health interventions, using smartphones and text-messaging, have seen a surge in development and use (5). These tools can make interventions more accessible: people across a wide range of socioeconomic groups own smartphones in the US, and ownership continues to increase globally (6). The percentage of Latinx individuals that own smartphones is currently 85%, comparable to Blacks (83%) and non-Hispanic whites (85%), and slightly higher than low-income Americans (76%) (7). Although ownership rates are high, disparities in digital literacy and data plan limits persist for ethnic minority and lower-income individuals (8). These factors necessitate user friendly and low data solutions.

The field of digital health has seen an increase in interest in conversational agents, or chatbots, to help individuals pursue healthy lifestyles (9). Chatbots for behavior change can inform and educate, check and monitor symptoms, and improve mental health through Cognitive Behavioral Therapy (10), motivational interviewing, and other therapeutic modalities (11). Chatbots can save costs, guard anonymity, and personalize content (12). Since chatbots can communicate with text-message or voice dialogue, lower digitally skilled users can easily engage (13). Finally, chatbots using natural language processing may be more powerful than one-way messaging because individuals can interact through a natural form of communication (14).

However, most health chatbots may be unsuitable for lower-income and ethnic minority individuals including Latinxs. Most health promotion chatbots offer only English as a communication language (15) despite over 40 million people in the U.S. speaking Spanish at home (16). For example, a systematic review on health chatbots (including general health and mental health) found that out of 45 chatbot studies, only one study used a Spanish chatbot (17). Similarly, our previous review on conversational agents for physical activity identified only a handful of conversational agents for mobile delivery through apps and text messaging, and no Spanish conversational agents (15). Further, digital solutions are not often specifically designed for women (18), especially ethnic minority women, who generally have worse health outcomes than white women (19). Ethnic minority women are underrepresented in the design and testing of digital health tools (18). Because of this and the lack of adequate digital skills training, marginalized groups may underuse these interventions (20), even if health technologies target them.

Thus, developing health chatbots for marginalized populations, including low-income women and Spanish speakers, is challenging but crucial and has not been attempted often enough. We developed a prototype text-message based physical activity conversational agent for low-income English and Spanish speaking individuals, based on principles of Behavioral Activation (21), Motivational Interviewing (22), Acceptance and Commitment Therapy (23), and Solution-Focused Brief Therapy (24). We designed the prototype chatbot to help users clarify their values and motivations for physical activity, set goals and plans, and overcome exercise barriers.

The aim of this study was to understand whether low-income English and Spanish speakers want to interact with conversational agents, what their health priorities are, and how we should design these tools to meet their needs. We report the results of qualitative interviews in low-income Spanish and English speaking women, majority Latinas, during testing of our prototype chatbot, a Wizard of Oz study, and a co-design workshop conducted in Spanish. We originally opened our study to all genders, but received interest only from participants who identified as female. We recommend best practices for researchers interested in developing chatbots for low-income women in English and Spanish.

## Materials and Methods

### Participants

We recruited participants through SNAP-Ed (Supplemental Nutrition Assistance Program Education) in California (Alameda County) and Tennessee (Shelby County). We consider participants low-income as the intended audience for SNAP-Ed is SNAP recipients (who are at or below 200% Federal poverty guidelines) and other low- income audiences who are at or below 185% Federal poverty guidelines.^1^ SNAP-Ed extension partners distributed English and Spanish flyers and invited participants in their healthy eating groups to join the study. In addition, SNAP-Ed posted our English and Spanish flyers on their Facebook pages. We included participants 18–65 years old, English or Spanish speaking, who owned a mobile phone and desired to be more physically active. This study was approved by the University of California Berkeley Committee for Protection of Human Subjects (CPHS, ref: 2020-05-13271).

### Conversational Agent Technical Development

**Figure 1:**
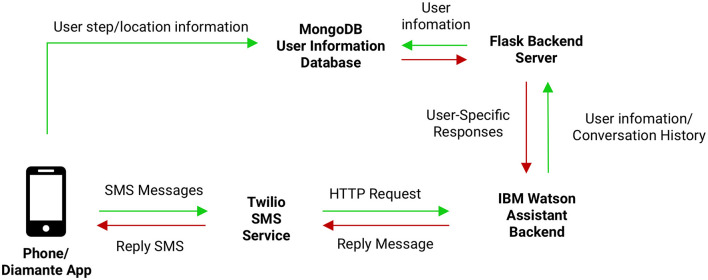
We used a front end SMS interface for communication, through the Twilio communication service, and a backend conversational agent using a cloud-based tool, IBM Watson Assistant, for developing and managing conversational flows. We leveraged a custom server for collecting record data and managing scheduled tasks, through a backend REST API implemented in Python Flask, with a MongoDB database.

We used a front end SMS interface for communication, through the Twilio communication service^2^, and a backend conversational agent using a cloud-based tool, IBM Watson Assistant^3^, for developing and managing conversational flows. We leveraged a custom server for collecting record data and managing scheduled tasks (see Figure 1), through a backend REST API implemented in Python Flask^4^, with a MongoDB^5^ database. Our dialog tree consists of over 150 conversational elements, with over 10 million possible conversational pathways.

### Conversational Agent Flow Development

Our prototype conversational flows went through two iterations during the study. The first iteration included flows on core values, motivation for health behavior change, exercise goals, and activity planning. Midway through the study we added flows on weekly exercise plans and barriers to physical activity, and we addressed errors after receiving participant feedback. Examples of the dialogue flows are shown in the Supplementary Material.

#### Study Measures

##### Phase 1: Online Interview

We interviewed participants online between August 2020 and April 2021. After participants filled in an online informed consent form, they were directed to a short online questionnaire that assessed gender, age, socioeconomic status, depression, anxiety, perceived stress, and financial issues due to the COVID-19 pandemic. Within a semi-structured interview conducted via videoconferencing, we assessed opinions and knowledge of chatbots as personal health coaches, technology use, digital literacy, and privacy considerations of chatbots in general. We also asked participants what information and text-messages they would like to receive from chatbots, and how chatbots could help them remain active during social distancing measures.

##### Phase 2: Wizard of oz Procedure

After answering these exploratory questions, participants completed a 20 min text-messaging conversation with a simulated chatbot. Participants were told that they were texting an automated chatbot but were actually interacting with a second researcher who texted participants via a Google Voice phone number. This procedure allowed us to understand how a chatbot should ideally respond in conversations with humans (25). Participants were debriefed at the end of the interview about the Wizard of Oz procedure.

##### Phase 3: Chatbot Prototype

Participants texted with our chatbot prototype for 10–20 min. After the texting conversation, participants resumed the semi-structured interview via videoconference. Participants were asked about their opinions of the chatbot prototype's ease of use, usefulness, humanness, and sustainability, as well as their preference for the simulated chatbot vs. the automated chatbot. They were also invited to give recommendations for expanding the chatbot prototype's content and improving upon its usability. The semi-structured interviews were recorded via Zoom and audio-recordings were transcribed by a professional transcription service (3playMedia). Participants received a $40 gift card upon study completion.

##### Phase 4: Design Workshop

Midway through the study, two researchers facilitated a co-design session in Spanish via Zoom for Spanish-speaking and bilingual participants who had completed the online interview and tested the chatbot prototype. During the workshop, participants added ideas for chatbot use and design to a Google Jamboard (Supplementary Material). Participants were compensated an additional $40 for their participation.

### Analysis

We show descriptives of the clinical and demographic characteristics of the sample using means, medians and percentages where appropriate.

We applied a constructivist grounded theory approach (26) to our qualitative analysis of the semi-structured interviews. TCL developed an initial codebook in English, which AM and AJ used to develop a Spanish codebook. We also employed open coding to generate codes inductively. After all co-authors met to discuss emerging themes, we revised our codebook to incorporate inductively- and deductively derived codes that captured user feedback and attitudes related to the content and physical activity motivators. The transcripts were coded independently by TCL, MM, AJ, and AM using Dedoose, a qualitative software program (27).

The codebook was divided into two distinct parts corresponding to Phases 1 and 3 of the study. The first part of the codebook was designed to analyze participants' comfort level with technology prior to engaging with the simulated chatbot and our chatbot prototype. Data from Phase 1 of the study were tagged with the following codes: “Technology familiarity” and “Privacy and security concerns.” The second part of the codebook synthesized data about participants' opinions of our prototype chatbot after engaging in a 10–20 min texting conversation with it. Data from Phase 3 of the study were tagged with the following codes: “Ease of use,” “Satisfaction, usefulness, and humanness,” “Sustainability,” “Physical activity barriers,” “Content and usability recommendations.” We memoed on the data tagged with these codes to generate the themes we present in this paper.

## Results

### Participants

We included 10 Spanish- and 8 English-speaking women aged 27–41 years. Most participants (*n* = 16) were recruited by SNAP-Ed Educators. The majority were Hispanic/Latina (*n* = 14), 2 were White and 2 were Black/African-American. More than half were monolingual Spanish speakers, and the majority were born outside the US (>50% in Mexico). For most, paying for basics was hard or somewhat hard (*n* = 17). Over half of the participants had finished high school and/or college (*n* = 11). Most participants (*n* = 16) reported being in good health, and depression and anxiety scores were low overall (>2 indicates risk for depression or anxiety). Table 1 shows participant characteristics.

**Table 1:** Participant characteristics.

	**Overall (*n* = 18)**
**Preferred language**	***n*** **(%)**
English	8 (44.4%)
Spanish	10 (55.6%)
**Age**	**Mean (SD)**
	39.5 (6.01)
**Race/ethnicity**	***n*** **(%)**
Black/African-American	2 (11.1%)
White or Caucasian	2 (11.1%)
Hispanic/Latina	14 (77.8%)
**Employment**	***n*** **(%)**
Full time (more than or equal to 35 h)	3 (16.7%)
Part time (<35 h)	1 (5.6%)
Homemaker	10 (55.6%)
Unemployed	2 (11.1%)
Retired	1 (5.6%)
Other	1 (5.6%)
**Education**	***n*** **(%)**
Never went to school	1 (5.6%)
Between 1st and 5th grade	2 (11.1%)
Between 6th and 8th grade	2 (11.1%)
Some high school	2 (11.1%)
High school graduate or “GED” degree	3 (16.7%)
Some college or technical school	1 (5.6%)
College graduate	5 (27.8%)
Graduate degree	2 (11.1%)
**Paying for basics (e.g., food, housing, medical care, and heating) is:**	***n*** **(%)**
Very hard	4 (22.2%)
Somewhat hard	13 (72.2%)
Not hard at all	1 (5.6%)
**Born in the US**	***n*** **(%)**
	5 (27.8%)
**Country of birth (if not US)**	***n*** **(%)**
El Salvador	2 (11.2%)
Guatemala	1 (5.6%)
Mexico	9 (50.7%)
Peru	1 (5.6%)
**Self reported-health**	***n*** **(%)**
Fair	2 (11.1%)
Good	10 (55.6%)
Very good	3 (16.7%)
Excellent	3 (16.7%)
**Impact of COVID-19 (1** **=** **completely disagree, 5=** **completely agree)**	**Mean (SD)**
I am running into financial issues	2.67 (1.37)
I feel more lonely	2.00 (0.970)
I feel more stressed	2.78 (1.48)
I feel more anxious	3.00 (1.41)
**Psychological measures**	**Mean (SD)**
Depression (PHQ-2, range 0–6)	1.06 (1.06)
Anxiety (GAD-2, range 0–6)	1.67 (1.28)

*PHQ-2, Patient Health Questionnaire, 2-item scale; GAD-2, Generalized Anxiety Questionnaire, 2-item scale*.

### Qualitative Findings

We divided the qualitative findings from the semi-structured interviews in Phases 1 and 3 into English and Spanish speaking participant feedback. Here we describe the main results. Additional quotes from participants can be found in the Supplementary Material.

#### Phase 1 Findings (Prior to Chatbot Conversations)

##### Technology Familiarity

###### English-Speaking Participants.

Most English-speaking participants had been sending and receiving text (SMS) and multimedia (MMS) messages for years, primarily through their phone's default messaging app but also through other messaging apps (e.g., Facebook Messenger, Google Hangouts, WhatsApp).

Texting familiarity: “*I want to say over 10 years...15 years I've been texting, forever.”*

Some participants reported also using their mobile phones for health purposes, such as diet and activity tracking (most commonly through Apple's Health app).

Health apps: “*I did have a fitness app… There's, obviously, the iPhone's health app, in general, for step counting. And my work handed out a health app, and I purchased Zombies, Run!*”

The majority were unfamiliar with the term “chatbot,” but said to have encountered a chatbot when the researcher gave examples–e.g., for food ordering and delivery, banking, internet service, and health care.

Chatbot experience: “*I think I might have [interacted with a chatbot]. Comcast, you know the Help Center? Yeah. I texted them and then I got a response. And then I texted something else, and I got another response. And at the end, they sent me a link to see if that could help*.”

English-speaking participants' initial perceptions of chatbots varied: some were optimistic about the potential uses of chatbots for streamlining interactions between individuals and services (e.g., “*I have no problem with it, especially with knowing the actual human technology that's guiding the technology behind it…I know that artificial intelligence is growing*”).

Others expressed doubt (e.g., “*I know that the world is evolving, and computers are being used for all types of things. But in general, I don't really like it*.”).

Some were skeptical, e.g., “*I think it's going to be frustrating. When I call to make a payment, I do it, and there's no one on the other side. And I ask a question, and [I] get something else.”*

###### Spanish-Speaking Participants.

Besides text-messaging, calling, and social platforms like Facebook's messenger, WhatsApp, Instagram, many Spanish speaking participants also used their phone for basic services.

Common uses for phones: “*I use it a lot (default texting app), for the bank, for my close family... And the phone…for my basic services: electricity, gas, water, insurance, all that…That's what I use it for more than anything else*.”

Participants used apps like YouTube as a resource for physical health, mental health, and healthy food recipes and followed doctors' advice on Facebook and YouTube.

Fitness resources: “*I like to watch some videos on YouTube...there are some exercises that I like... They are like boxing with cardio or something... I also like the yoga ones.”*

Most Spanish-speaking participants were unfamiliar with the term “chatbot,” until given examples of automated machines (e.g., a phone answering machine).

Even though all had been using text-messages, some participants did not know how to send a new text message to a new number. Additionally, most participants were unfamiliar with video conferencing software and required help from a researcher and their children or partner to navigate Zoom and initiate a conversation with our chatbot.

##### Privacy and Security Concerns

###### English-Speaking Participants.

Many English-speaking participants were concerned about privacy and were hesitant to share personal information such as their name, images of their face, and location. They were concerned about sharing their location and the chatbot recommending unsafe locations.

Location recommendations: “*They [the chatbot] say you can go to this park. but I'm going to be concerned if the park is not being cleaned often, or who gets there. Who goes there? And even though the chatbot is going to tell me this place is safe for you to go, I'm not sure if I'm going to give it a try*.”

###### Spanish-Speaking Participants.

Privacy and security concerns mostly stemmed from limited understanding of chatbot technology. Participants' questions included: “*Do people see what the chatbot is doing? Do people review the messages? Can the chatbot see what I am doing?”*

Additionally, participants were concerned about sharing location data:

“*I would say that [sharing location] is fine, but on the other hand it would be wrong because I would feel like they know where I am and where I'm going and everything. I would feel like I was being watched.”*

#### Phase 3 Findings (After Testing Prototype Chatbot)

##### Ease of Use

###### English-Speaking Participants.

Participants universally appreciated the chatbot's quick response times. As one participant explained, “*the thing I didn't like about the [Wizard of Oz] was that the response time wasn't as fast as the [chatbot]*.”

Others commented that they felt the chatbot “*was going more around the question and not really giving me any solutions.”*

Usability issues were a common complaint. Many participants had to restart the conversation after the chatbot failed to recognize their input.

Restarting conversations: “*I put in a lot and they sent me back: ‘Hi, I am your physical health companion.' So they sent me, again, the same message that they sent me at first*.”

###### Spanish-Speaking Participants.

Participants found the overall experience positive. After the researchers gave coaching and instructions, most participants regardless of literacy level were able to send and receive text messages. After teaching a participant how to create a new message, our team asked the participant if she found the chatbot session to be difficult, to which she replied: “*No, it's easy, I didn't find it difficult myself.”*

One participant was unable to reply to the chatbot, as she had difficulties reading, writing and typing. When she needed to send a message, she would use the voice input feature on her phone instead of typing out the message.

##### Satisfaction, Usefulness, and Humanness

###### English Speaking Participants.

Overall, English-speaking participants were satisfied with their interactions with the chatbot. Participants described the chatbot as “*really great,” “pretty conversational,” “very responsive,”* and “*very innovative*.” One participant stated that the chatbot could be her support person, just like her friend. Some participants preferred texting with the chatbot over texting with a researcher. One participant noted that “*there weren't any grammatical errors in the [chatbot] responses*,” as opposed to the human responses. Another user stated, “*I think this was a little bit better, the second one. Even though I had a little more problems, it was a little more specific or more detailed*.”

Participants did not find the chatbot very humanlike, especially when they compared it to their interactions with another researcher via the Wizard of Oz procedure.

Personalization: “*It seemed like the first one was more conversational–it's more personal, kind of, than the other one. It was just like...answer this question on the second one, to get to the next thing*.”

Most participants recognized and accepted the limitations of the chatbot.

Satisfaction: “*I mean this is cool. Like I said, I would prefer to talk to a live person, but if that's the next best thing, then it's pretty cool*.”

###### Spanish-Speaking Participants.

Spanish-speaking participants mentioned the chatbot could give them a quick confidence boost, and help address problems when friends or family members are unavailable. Messages of encouragement and reminders were among the features most liked/requested.

Encouragement: “*And I get a reminder (from the chatbot) and I need to get back to you (the chatbot) and I can say: ‘oh, I am sorry, I am not feeling well today.' And I receive a message saying ‘no, it is all right, you can do it!' In other words, a message of encouragement would be very nice.”*

Another participant pointed out that humans can get tired of trying to encourage one another, or because of cultural norms, might not be the most supportive.

Support system: “*Yes, because many times there are people who are very depressed and their relatives don't go to her because they can't go to their house or things like that...that someone (the chatbot) sent them a message I imagine they must get motivated. To say, ‘wow, somebody remembered me.”'*

In contrast to the English-speaking participants, most Spanish-speaking participants found their interactions with both the Wizard of Oz chatbot and the automated chatbot to be human-like. One participant stated that she felt like she was texting a person, “*I felt comfortable with the application (chatbot) because it's like I was talking to someone, but it wasn't someone.”*

Participants could not differentiate between the researcher and the chatbot and would often ask how the chatbot could be a machine.

##### Sustainability

###### English-Speaking Participants.

When participants were asked how long they would use the chatbot for, answers ranged from 1 month to indefinitely. Some participants considered engaging with the chatbot daily (and even multiple times throughout the day) for workout ideas, exercise plans, and new exercise knowledge.

Continued use: “*As long as I'm exercising or if I set a goal for like a month or two months, I think it would be great to have at least reminders or stuff like that, during the whole month, or during these two months. So it would be useful*.”

One participant found the chatbot to be more sustainable than an app or activity tracker because “*you're talking to somebody to help you work through things and think through things, and apps aren't really going to do that*.”

Some participants felt that the chatbot would lose its novelty over time, especially if its responses became repetitive or unhelpful.

Repetitiveness: “*If they don't keep up with my goal and they keep asking the same things and not moving on to the place that I am after, probably, two months from now, that will make me stop using it*.”

###### Spanish-Speaking Participants.

Most participants stated that they would continue to interact with the chatbot if it remained free. They also shared that they would primarily use it when they need suggestions for healthy eating and new exercises.

Seeking advice:“*I think when I need help. Like before exercising, ask him[the chatbot] about some exercise...and also about nutrition.”*

Participants also mentioned wanting to invite their family to engage with the chatbot, e.g., “*Well, not only me, I think that even the children would like it to motivate them, something like that*.”

##### Physical Activity Barriers

###### English-Speaking Participants.

The majority of our participants expressed a desire to become more physically active, but also shared several barriers that prevent them from achieving their exercise goals. These barriers include work (“*This month I haven't been doing as much [exercise] because I've been a little bit busier with work”)*; lack of companionship and social support (“*It's not motivating to start exercising by myself”)*; lack of routine; injury; gym closures; and weather *(e.g., Winter is also coming, so it's like people are going to be inside even more)*.

Participants found that their daily lives were impacted due to caregiving duties. “*I have three girls of different ages and they all need something from me all the time. So I don't have much time for myself. So if I find a time to do something that I like, I just do [my errand] instead of like, stop because the park is on my way*.”

###### Spanish-Speaking Participants.

Most physical activity barriers for the Spanish-speaking group overlapped with English-speaking participants. One participant shared how she was impacted by the stay-at-home-orders.

Fear of infection: “*I felt very scared because the news came like a bomb...people are dying and they are getting infected, so I already imagined that we were going to get infected...it was like a trauma for me, the truth is that I stopped doing my things from one day to the next.”*

For a few participants, physical injuries limited their physical activities.

Bodily pain:“*I suffer a lot from my lower back and I like to see exercises that help me with that...I really like yoga.”* Participants identified personal physical activity recommendations as a benefit of technology like our chatbot prototype.

#### Content and Usability Recommendations (English and Spanish Participants)

Participants gave specific recommendations to expand upon the program content and chatbot functions. We show the main recommendations in Table 2.

**Table 2:** Specific recommendations for content and functions.

**Content**	1) Providing pictures and videos of suggested exercises
	2) Including healthy eating tips
	3) Sending periodic exercise reminders throughout the day
	4) Sending daily fitness tips
	5) Describing health benefits of physical activity in more detail
	6) Setting milestones for personal fitness goals
	7) Including weather- and location- specific exercise suggestions (e.g., activities that can be done in the house when it's raining outside)
**Functions**	1) Allowing for alternative text input methods (e.g., integrating voice-to-text)
	2) Allowing users to input their own barriers to physical activity
	3) Giving users the option to select answers from drop-down lists
	4) Integrating more opportunities for social connection (e.g., a chat room or integration to existing social platforms)

#### Co-design Workshop in Spanish

Background information is shown in the Supplementary Material. Here we report the main results.

Participants mentioned that they would like our chatbot to help with activities other than exercise, including finding health information, healthy recipes, COVID-19 vaccine information, and anti-stress tips. If they could create their own ideal chatbot, they would like it to also help them with payment reminders, advice about healthy environments, exercise and cooking tips, and recommendations for visiting new places. Finally, they would design chatbots that help young people study and go to university, and help with parenting, including activities for children.

## Discussion

We describe the opinions of low-income English- and Spanish-speaking women on health chatbots, and their experiences with our prototype physical activity chatbot. Overall we find that participants were largely unfamiliar with chatbots, and were initially skeptical of their use. After testing our prototype, most users felt positively about physical activity chatbots. They were concerned about the privacy of their personal information, especially involving location. Users indicated wanting a more comprehensive chatbot system that provides daily exercise goals, tips, health information and healthy food/cooking options. We found differences between English and monolingual Spanish speakers in exercise app use, digital literacy, and the wish for family inclusion. Below we provide specific recommendations for designing health chatbot interventions for this population.

### Chatbots, Despite Being Machines, Can Provide Physical Activity Support

Participants were initially skeptical of communicating with a machine. After testing, most commented that the chatbot could give them support and tips when family and friends failed to provide it. Both English- and Spanish-speaking participants found the chatbot to be motivating, similar to a friend that encourages you to exercise. Previous work on automated text-messaging for mental health, also highlighted that Spanish speakers in particular perceive social support from automated text-messages (28). Thus, even if participants know they are talking to a machine, the chatbot could still provide emotional support, particularly when others are too busy or unwilling to provide it.

### Pay Attention to Linguistic and/or Cultural Differences Between English and (Mono-Lingual) Spanish Speakers

In contrast to our English-speaking participants, Spanish-speakers used few health apps. Instead, they used social media such as Youtube to find health information. Further, Spanish-speakers generally had lower tech literacy and higher barriers to participating in health tech studies, in line with results from previous studies (8, 29). Several Spanish-speaking participants needed help from the researchers to set up a Zoom meeting and initiate a conversation with the chatbot. For inclusive digital health, this support should be a standard component of the research visit. Finally, though many participants indicated a wish to add a social component to the chatbot, Spanish speakers in particular wanted their family members (including their children) to be able to engage with the chatbot. Thus, differences were found mostly in exercise app use, digital literacy, and the wish for family inclusion.

### Use Simple Text-Based Chatbots, Allow Voice Communication, and Provide Tech Support

Our findings combined with earlier work (30) demonstrates an advantage of a text-based chatbot rather than an app-only interface. Most participants were familiar with texting, but not all were frequent app users. Other work in Latinx parents also suggested that using tools that participants are already familiar with for chatbots, such as Whatsapp will be most effective. For participants with lower reading, writing and tech literacy, voice interactions could further increase the usability of chatbots. For example, a user co-creation study showed that older adults, who generally have lower digital literacy, prefer voice based physical activity chatbots to text based (31). The authors suggested that voice is a powerful modality for encouraging motivation among those who struggle with new technologies.

Participants also indicated they preferred short and easy-to-understand messages. In line with other work (32), we recommend that the reading level should be no more than 8th grade (13–14 years) literacy. Finally, technical issues (e.g., chatbot struggled to understand answers outside of a template) impeded smooth communication. Flexibility of bot response, dialogue length, dialogue structure, and chatbot personality are general technical challenges of chatbot design, which need to be addressed and improved in future work to avoid user frustration and drop-out (33).

### Increase Transparency About Data Collection

Most users were concerned about sharing personal information, particularly their location. Users felt more comfortable with data sharing if they understood the reason for that data collection (e.g., giving more personalized tips). Informing users of the reason behind requesting information may make them feel more comfortable and willing to use the technology.

### Consider Designing Comprehensive Chatbots to Help With Health and Non-health Activities

Most participants indicated they wanted help with healthy eating and finding reliable health information (including COVID-19 related information). Further, many Spanish speakers suggested chatbots should include the rest of their family into healthy living, and also provide parenting tips. Several participants envisioned chatbots also helping with practical activities such as banking, cooking and parenting. Researchers could consider comprehensive wellness chatbots that can connect individuals to other non-health related services. One drawback of this approach is the more functions a chatbot includes, the more difficult it becomes to design the chatbot, and to measure its effects (34). Factorial designs such as the multiphase optimization strategy might be helpful in this case to measure the effects of various chatbot components (35).

### Consider Adaptive Chatbots to Keep Novelty

Users expressed concerns of boredom with chatbots over time if functions and recommendations remain unchanged. In our previous systematic review, we found that when program content was repetitive, users were more likely to disengage from chatbots (15). Most conversational agents included in our review lacked personalization and only communicated through multiple-choice responses. Studies also neglected to discuss safety and privacy issues, and few conversational agents acted on users' mentions of injuries, pain, or mental health symptoms. To increase engagement, chatbots should be adaptive (36–38), for example change the goals and tips based on participant behavior, and add new recommendations, weather and location specific tips and videos. Some participants also mentioned a wish to include their own exercise barriers, and be connected to others through a chatbot. Options would be to allow for more user input, and add peer support, or a human health coach when the chabot fails to provide adequate guidance.

### Partnering With Community Organizations and Co-creation

Partnering with local community partners proved to be essential for recruiting typically underrepresented participants into our study. When SNAP-Ed health trainers recommended our study, participants were much more inclined to participate. Through flyers on the Facebook pages of SNAP-Ed, very few participants enrolled (only 2, both white and non-Spanish speaking). Community partners should not only be involved in the recruitment process, but also in the dissemination of health technology innovations. Our co-creation session brought unexpected participant preferences and wishes, which were useful in developing subsequent versions of our chatbot. Co-creation also helps to prevent “bad design,” e.g., designing an intervention our target group won't use (39, 40). Thus, engaging both community partners and participants in the design, testing and dissemination of interventions can increase the likelihood that participants will use and benefit from health chatbot interventions.

### Limitations

We included low-income women who had already demonstrated a vested interest in their health by joining the SNAP-Ed healthy nutrition program. Women were paid for their participation in the study, which may have impacted their responses and the outcomes of this study. These findings may therefore not generalize to a less motivated group who will not receive compensation. Further, the number of participants was low. This study must be considered a pilot. We divided our findings based on English- and Spanish-speaking women. However, among the English-speaking women, half were bilingual Latinas (also spoke Spanish). Cultural differences may therefore be small, though the English-speaking participants may be more acculturated. We observed differences between English- and mono-lingual Spanish-speaking participants mostly related to digital literacy and app use.

### Future Steps

This research confirms that participants are interested in talking to computer-based agents about social and physical health issues. Future work should test more finalized chatbot applications in this population in larger studies, with a subsequent user-testing phase of a more finalized version of the application and ultimately in a randomized clinical trial. Most systems are currently too inflexible to personalize care because they use rule-based responses. Increasing personalization, creating chatbots that can respond flexibly, and advancing health equity by reaching marginalized populations should be major goals of future chatbot research. By tailoring this intervention to low-income Spanish- and English-speaking women-who can greatly benefit from mobile health applications but for whom they are often not designed-researchers can contribute to improving health and (digital) health equity.

### Conclusion

Low-income Spanish- and English-speaking women are interested in using chatbots to improve their physical activity and general health and feel supported by these tools. Issues that researchers should take into account when designing chatbots for this population include digital literacy, app familiarity, linguistic and cultural issues, privacy concerns and novelty and flexibility. Using co-creation techniques and involving community partners will increase the likelihood that health chatbots will be effective. Future work should focus on personalization, co-creation, and ensuring health equity through digital innovations.

## Data Availability Statement

The raw data supporting the conclusions of this article will be made available by the authors, without undue reservation.

## Ethics Statement

The studies involving human participants were reviewed and approved by University of California Berkeley Committee for Protection of Human Subjects (CPHS). The patients/participants provided their written informed consent to participate in this study.

## Author Contributions

CAF wrote the first draft of the article. TCL, MM, AJ, and AM conducted the user testing and the qualitative coding. DC developed the technical infrastructure of the chatbot. AA and JC provided feedback throughout the study. All authors contributed to the writing of the final version.

## Funding

This study was supported by pilot grants from the Center for Information Technology Research in the Interest of Society (CITRIS) and the Center for Technology, Society & Policy at the University of California Berkeley, and a grant from the Latinx Center of Excellence in Behavioral Health at UC Berkeley. The Latinx Center of Excellence in Behavioral Health was supported by the Health Resources and Services Administration (HRSA) of the U.S. Department of Health and Human Services (HHS) as part of a CARES Act award totaling $150,000 with 0% financed with non-governmental sources. For more information, please visit HRSA.gov.

## Author Disclaimer

The contents are those of the author(s) and do not necessarily represent the official views of, nor an endorsement, by HRSA, HHS, or the U.S. Government.

## Conflict of Interest

The authors declare that the research was conducted in the absence of any commercial or financial relationships that could be construed as a potential conflict of interest.

## Publisher's Note

All claims expressed in this article are solely those of the authors and do not necessarily represent those of their affiliated organizations, or those of the publisher, the editors and the reviewers. Any product that may be evaluated in this article, or claim that may be made by its manufacturer, is not guaranteed or endorsed by the publisher.

## Footnotes

^1^ https://www.fns.usda.gov/snap/snap-ed

^2^ https://www.twilio.com/

^3^ https://www.ibm.com/cloud/watson-assistant

^4^ https://flask.palletsprojects.com/en/1.1.x/

^5^ https://www.mongodb.com/cloud
