# Supplementary material for: Conversational Physical Activity Coaches for Spanish and English Speaking Women: A User Design Study
Source: Front Digit Health. 2021 Oct 8;3:747153. doi: 10.3389/fdgth.2021.747153 (PMC8531260; doi:10.3389/fdgth.2021.747153)
Supplement: Supplementary file 1 [file Data_Sheet_1.docx]

Supplementary material Figueroa et al.

**Content:**

[**Examples of Conversational Flows**](#_heading=h.30j0zll) **[1](#_heading=h.30j0zll)**

[**Additional Quotes from Participants**](#_heading=h.1fob9te) **[4](#_heading=h.1fob9te)**

[**Co-Design Workshop: Charla Comunitaria**](#_heading=h.3znysh7) **22**

[Translated Jamboard Session](#_heading=h.tyjcwt) 26

# Examples of Conversational Flows

Hi! I'm your physical health companion. I can help you set health goals, send you reminders, and motivate you. First, I need to learn more about you. What is your name?

Nice to meet you, ! Health goals are different at every age. How old are you?

Now I’d like to learn what is important to you by asking you about your core values. Do you know what core values are?

No problem! Core values are the things that are most important to you. They guide how you live your life. Would you like some examples of core values?

A few examples of core values are achievement, community, family, friendship, fun, happiness, health, independence, knowledge, passion, peace, and success. Would you like some more examples?

Now that I know your core values, I can help you set and achieve goals later.

Health is a core value for many people. On a scale from 1 to 10, with 1 being not important and 10 being the most important, how important is your health to you right now?

Good to know! It sounds like you care about your health. Even if health isn’t the most important to you, improving your health can help you achieve other goals. Do you want to know how physical activity can improve how you feel?

You said that [user’s core value: community, family, friends, relationships, etc.] is one of your core values. Does support from friends or family motivate you to exercise?

Great! Support from others can help us reach our goals. Do you enjoy exercising with people?

That’s great! We can plan for you to exercise with others. Would you like to hear ideas for what you can do together?

Some activities that are good for groups are hiking, biking, walking, dancing, tennis, basketball, and taking a fitness class together.

You said that [user’s core value: fitness, health, wellbeing, etc.] is one of your core values. Is improving your health one of your main reasons for exercising?

Excellent! What do you want to work on? Maybe you want to build muscles, be more flexible, get faster, exercise longer, lose weight, or stay free of disease.

Thanks for sharing! There are many exercises you can do to [user’s answer: improve endurance, build muscle, etc.]. Would you like some tips?

Regular stretching will help you be more flexible and exercise safely. Try to hold the position for 10 to 30 seconds. Remember to keep breathing during the stretch!

Some good exercises for weight loss are running, walking, biking, swimming, and lifting weights. Slowly start to exercise more. Weight loss takes time. Do not feel bad if you don’t see change right away!

You said that [user’s core value: achievement, passion, responsibility, etc.] is one of your core values. Does [core value: achievement, responsibility, etc.] motivate you to exercise?

That’s great! Tell me more. How has [core value] helped you in other areas of your life?

When were you able to do something because of [core value: passion, sense of responsibility, etc.]?

How can physical activity improve your life? Think about how you feel when you plan to exercise and go through with it.

Thanks for sharing! Sounds like [core value] has helped you with many things. Remember that as you start your health journey!

You said that [user’s core value: energy, excitement, happiness, peace, etc.] is one of your core values. Are you motivated to exercise to improve your mood?

Great! Try to choose activities you enjoy doing and exercises you can do for a long time. What activities do you enjoy doing?

Those are all great! Would you like to hear about exercises that others enjoy?

Jogging, swimming, and biking get your heart rate up and increase endorphin levels. This makes you feel happier.

Yoga calms the mind and body. It teaches you to relax and let go of stress.

Hiking makes you feel peaceful and relaxed. Being outdoors is a good break from stress.

Dancing combines music and moving. It can help you feel happy and energetic.

Try exercising alone and with others. Get creative and keep things fresh!

Now let’s build on your motivation and set a goal for your exercise this week.

Let’s make a plan to reach your goal. What exercise do you do now?

How many minutes per day do you exercise now?

Got it! I recommend you exercise 3 days a week for 30 minutes or more. Let’s plan your exercise for this week. What days would you like to exercise?

Nice! Let’s start with [*first day that the user listed*]. What exercise would you like to do? How many minutes will you do that for?

Now let’s talk about barriers to exercise. What are some things that might stop you from reaching your goal?

Time is a common barrier. A lot of people say they do not have enough time in the day for everything. Do you feel that way?

Thank you for sharing. What are some things you *have* to do every day?

Are you able to spend *less time* on any of the things you listed?

Even cutting 10 minutes from 1 activity can give you enough time for a short walk or workout. Another option is to fit exercise into your home routine. Do you want to hear some ideas?

Stretch while reading. Do sit ups or push ups while watching TV. Do lunges while vacuuming, and do squats while cooking. Stand up when talking on the phone. Do you think you could do some of these things?

Does low mood or low motivation prevent you from exercising?

I’m sorry to hear that you feel down sometimes. There are a few things you can do that may help. First, let’s think about why exercise is important to you. How would reaching your exercise goal improve your life?

That’s good to know. Now please tell me about a time when you felt good during or after exercising.

Thanks for sharing. How do you think you will feel if you do exercise today?

Does pain, soreness, or a health condition prevent you from exercising?

You are not alone - pain is a common barrier to exercise. Soreness after a hard workout is normal, but other types of pain can be more of a problem. How would you describe the pain that you feel?

Are there exercises that are less painful for you?

Do you want to hear about some exercises that have less impact on the body?

Swimming is a great low-impact, full-body activity. Being in water takes the pressure off your joints and muscles. Yoga helps build a strong core and improve balance without putting stress on your joints. Walking is not as hard on your bones as running but still lets you stay active. Can you try one of these activities?

You can also think about picking different exercises based on where you are feeling pain. For example, if your legs are feeling sore, you can try doing some upper body exercises like push ups, sit ups, and lifting weights. Does that make sense?

# Additional Quotes from Participants

Quotes in English and Spanish. Spanish quotes include the English translations below. Original responses are unedited; only their translations are edited to convey an accurate translation.

**Experiences with technology, apps and chatbots (before testing our prototype)**

***English-speaking participants:***

- To be honest, I don't like [chatbots]. I mean, I'm being transparent. I don't like talking to a computer. I really don't. I mean, I know. it's probably efficient as far as trying to save me answering the same questions or whatever just for time or whatnot. But I really don't-- I don't like it. I mean, I know that the world is evolving and computers are being used for all types of things. But in general, I don't really like it.
- But what will happen if the functionality of these chatbots-- I mean, what if it's not programmed to answer all the questions? That's what frustrates people. I mean, I'll ask a question, and they'll keep asking me the same thing. And I'm like, a representative, please. And I'll just-- and it's just frustrating. And I hit 0, just 0, a representative. And they'll say, oh, that's an invalid number. And then you keep hitting 0. And then they'll immediately transfer you to a representative. It's like, OK, you're not listening.
- I don't think [chatbots are] preferable, right? I'd rather talk to a live person if I could, right? But I think it's another option. If that's not available, then what's the next best thing? So if that's the next best thing, it's fine. I think it's helpful if you don't really need to speak with somebody about something. But again, it's minimal stuff, like, “What are your hours?”. Things like that I mentioned before. So I think it depends on what the situation is, but I think it's fine. It does its purpose, right? Technology is-- that's what it is. Technology has all these ways to make us more efficient and to be more helpful and stuff like that. I don't know, I guess I have mixed feelings. Like I said, I prefer to talk to a live human, but if this is the next best thing that's what you're going to get, it's better than not getting anything at all. I don't know. I don't know if it really answered your question.
- I have Alexa. And I use Siri in the car when I'm driving all the time. So I have no problem with it. Especially with knowing the actual human technology that's guiding the technology behind it. I don't have a problem with it. I know that the artificial intelligence is growing. So if it'll help, yeah, I don't have any problem with it.
- No, I don't know what a chatbot is. I have no idea.
- It's good, but I feel like we always have more questions or concerns depending on the situation. But sometimes it is good to have easy access to answers when we have questions. Sometimes [chatbots are] OK, but sometimes I feel like I need someone to answer my question. Like, if I have any different concern or I need to talk with someone to explain better to me something.

***Spanish-speaking participants:***

- Ah, OK. Tengo el Facebook, el Messenger, tengo el, este, ¿cómo se llama? Tengo Chase Mobile, tengo el Citibank Mobile, tengo el, cuál sería, el OfferUp, tengo-- ¿Qué otras tengo? ¿No sé si el Mapas sería aplicación? El Google Maps, tengo el Gmail. ¿Sería aplicación?
  - Ah, OK. I have Facebook, Messenger, I have the, this, what's it called? I have Chase Mobile, I have the Citibank Mobile, I have the, what would it be, the OfferUp, I have-- what other ones do I have? I don't know if Maps would be an app? Google Maps, I have Gmail, would it be an app?
- Tengo Pandora, tengo Yahoo, tengo Instagram, tengo Shazam, se llama esa aplicación. Tengo PowerSchool para la escuela de los niños. Tengo otro que se llama TalkingPoints que es para la otra escuela de mis hijos. Y creo que son las únicas que tengo. Tendría alguna otra, pero no, no sabría ahorita como estoy usando mi teléfono, pero sí, casi son la mayoría de las que uso.
  - I have Pandora, I have Yahoo, I have Instagram, I have Shazam, I think that's the name of that app. I have PowerSchool for the kids' school. I have another one called TalkingPoints which is for my kids' other school. And I think those are the only ones I have. I would have some others, but no, I wouldn't know right now as I'm using my phone, but yeah, they're pretty much most of the ones that I use.
- Oh, pues cuando veo eso a mi me gusta ver las en-- O sea, también tengo la aplicación de YouTube. Me gusta mirar algunos videos en YouTube.
  - Oh, well, when I watch that I like to watch that on-- I mean, I also have the YouTube app. I like to watch some videos on YouTube.
- Yo sufro mucho de la parte de la espalda baja y me gusta ver ejercicios que me ayudan en ese tipo de yoga. Me gusta. Y una vez traté una aplicación, no recuerdo cómo se llama, de cómo administrar el agua, cómo tomar el agua todos los días. Pero se me arruinó el teléfono que tenía y pues ya en el próximo teléfono, pues la verdad ya no la traté. Pero no recuerdo cómo se llama.
  - I suffer a lot from my lower back and I like to see exercises that help me in that type of yoga. I like that. And once I tried an application, I don't remember what it's called, about how to manage water, how to drink water every day. But the phone I had was ruined and then on the next phone, well, I didn't try it anymore. But I don't remember its name.
- Pero sí trato de buscar a veces, a veces, no te digo que diario, pero sí busco videos o cosas que me ayudan a la salud. Me gustan los remedios naturales y trato de buscar un poco.
  - But I do try to search sometimes, sometimes, I don't say daily, but I do look for videos or things that help me with my health. I like natural remedies and I try to search a little bit.
- Casi cuando busca así, tips de salud, así, ¿son en YouTube? Pues sí, la mayoría sí. En YouTube y a veces las busco en la página de Google.
  - So when you search like, health tips, like this, are they on YouTube? ‘Well yes, most of them are. On YouTube and sometimes I look them up on the Google page.’
- Entonces, ¿ahorita no utiliza ninguna aplicación para grabar cosas, como grabar su consumo de comida o sus ejercicios? No. Aplicación no. Lo que estuve haciendo en fuera, hacerlo manualmente en una hoja pegada en mi refrigerador.
  - So you currently don't use any apps to record things, like recording your food intake or your workouts? ‘No. No app. What I've been doing on the outside, doing it manually on a sheet stuck on my refrigerator.’
- Sí, sí me siento cómoda. ¿Y por cuánto tiempo diría que lleva haciendo eso, mandando y texteando? Mandando y texteando, pues, ¿qué será ya? Unos 10 años a la mejor. Sí, más o menos.
  - And how long would you say you have been doing that, sending and texting? ‘Sending and texting, well, what will it be now? Maybe 10 years. Yes, more or less.’
- Eso lo uso mucho, pues para lo que es el banco, lo que es así y pues lo que es mi familia cercana. Y ya para las otras utilizo, lo que es mi familia lejana, como el Facebook, el WhatsApp, el Messenger. Eso ya los utilizo para lo que es familia que está lejos, que no está aquí cercas. Y lo que es el teléfono, pues casi lo que es aquí en el área donde yo vivo y lo que tengo que hacer, por ejemplo, para mis servicios básicos, lo que es la luz, el gas, el agua, aseguranza, todo eso, el banco. Más que nada para eso lo utilizo.
  - I use it a lot [default text messaging application], for what is the bank, for what is like this and for what is my close family. And for the others I use, what is my distant family, such as Facebook, WhatsApp, Messenger. I use them for my family that is far away, that is not here. And what is the phone, well, almost what is here in the area where I live and what I have to do, for example, for my basic services, electricity, gas, water, insurance, all that, the bank. That's what I use it for more than anything else.
- Algunas aplicaciones me he dado cuenta de algunas, así como dices, algunas compañeras o familia, y algunas otras más que nada por parte de mi hijo. Y otras me aparecen cuando estoy allí navegando en Facebook, casi ahí es donde me han aparecido, como la de tomar agua o así. Me han aparecido como anuncio y dice pruébala y administra todo, consumo de agua y así. Y la mera verdad, pues como me aparece gratis, pues yo me voy allí al Google, no me acuerdo, al Google Store, algo así se llama.
  - Some applications I've been made aware of, so as you say, some of them, some of them from coworkers or family, and some of them mostly from my son. And others pop up for me when I'm there browsing Facebook, that's almost where they've popped up for me, like the one for drinking water or so. They have appeared as an advertisement and it says try it and manage everything, water consumption and so on. And the truth is, since it appears for free, I go to Google, I don't remember, to the Google Store, something like that is called.
- Y yo, la mera verdad, aparece gratis y yo trato de mirar. Porque todas las que me aparecen de qué valen algo, ahí sí no le sé mucho en ese aspecto en que dice que tienes que meter tu PayPal, que la tarjeta, o así. Ahí sí no lo sé mucho. Por eso yo ahí sí no me meto.
  - And I, the truth is, it appears for free and I try to look. Because all the ones [apps] that I see that are worth something, I don't know much about that aspect where it says that you have to enter your PayPal, that the [credit] card, or something like that. I don't know much about that. That's why I don't get involved there.
- ¿Ha tenido experiencias con esas aplicaciones que le piden su ubicación? Sí. Fíjate que sí. Y hay veces que cuando me piden mi ubicación, a veces yo las desecho, no me gusta a veces como poner mi ubicación. Sí. No me siento conforme o me siento segura, no, esté, cómo se llama, la desecho. La desecho.
  - Have you had experiences with those apps that ask for your location? 'Yes, I have. And there are times when they ask me for my location, sometimes I discard them, I don't like to put my location sometimes. Yeah. I don't feel comfortable or I don't feel safe, I don't, it's, what's it called, I discard them. I discard it[location sharing request message].’
- ¿Usted ha texteado a una máquina o no? Voy a decir que no, nunca lo he hecho.
  - Have you texted a machine or not? ‘I'm going to say no, I never have.’
- Pues, tengo varios tiempos usando mi teléfono, el mensaje. A veces recibo mensajes o así, ¿verdad? Pero sí tengo años usando para enviar mensajes...Como unos 16 años.
  - Well, I have several times using my phone and text. Sometimes I get messages or so, right? But I do have years using [the phone] to send messages...Like about 16 years.
- Solo le quería preguntar que, ¿si usted sabe qué es un chatbot? No, eso le iba a preguntar, que no sé qué es.
  - I just wanted to ask you, do you know what a chatbot is? ‘No, that's what I was going to ask, I don't know what it is.’
- Bueno, he bajado unos juegos para entretenerme....bajé uno también que es de palabras también porque quiero aprender. Y también bajé la del Facebook para el chisme.
  - Well, I downloaded some games to entertain myself.... I also downloaded a word game because I want to learn. And I also downloaded the Facebook one for the gossip.
- Y esto es nuevo para uno y, pues, con mi hijo le digo, ándale, hijo, ayúdame en esto, así. Pero sí, digo, no sé mucho de tecnología. Con mi hija también. Tengo una hija de 5 años que está en el kinder y es por Zoom también.
  - And this is new for me and, well, with my son I tell him, come on, son, help me with this, like this. But yes, I mean, I don't know much about technology. With my daughter too. I have a 5-year-old daughter who is in kindergarten and she is also in Zoom.

**Security concerns**

***English-speaking participants:***

- I don't really use [location sharing] because I don't feel safe.
- I can share, for example, if I use like a-- instead of my real name, Laura, I can use, say, watermelon. Use another name, fake name, and share my steps or things like that, I feel comfortable sharing but not with my real name or my face. Something like that.
- I'm not going to be comfortable sharing my location. I'm not going to be comfortable sharing probably not my date of birth, my social security number-- just basic general questions, just general, not something where it's going to be very specific. Oh, what is your date of birth? What is your social security number? What is your address? I'm like, it's just too much. And if I did share it, it has to be something in writing or something on a computer that will say, this information is 100% private. It will not be shared with other competitors. It has to be something that will ease my mind on my information being stolen because, you know, I just don't-- I don't want my identity-- I don't want to have to deal with identity theft.
- If I'm somewhere for 911 or whatever, if it's something that I know that's going to alert the police or something that I'm uncomfortable with, I wouldn't mind, if there's an emergency situation, sharing my location. It just depends on the circumstance and what's going on.
- I used location sharing with my family-- my husband and my children. You know, if I'm somewhere and they need to know where I am, they will know in advance. So I'm happy they know my location.
- I don't have like a permanent thing where it's showing my location. Most of them are like, if I open the app in this place, then you can see my location. Like even just for the grocery store, if I open it-- usually I open it if I go there. So it can tell that I'm actually there or nearby, if I open it. But I don't have any that are just completely aware of where I am.
- I hardly ever share [my location] with any apps, to tell you the truth. I only share it with Google Maps. That's all the location sharing I do. I feel kind of iffy about it.

***Spanish-speaking participants*:**

- Por ejemplo, una segura, pues es como cuando voy al banco y allí mismo me dicen baja la aplicación y vamos a abrirla ahorita. Y ahí mismo ellos me dicen cuando te aparezca, si andas en otro lugar y necesitas usar un banco cerca y tú abres tu aplicación y te aparece este sitio quiere usar tu, cómo se llama tu ubicación para poderte dar acceso a bancos cercanos, como que sí me siento segura porque hay alguien más que me lo está diciendo.
  - For example, a secure one, it's like when I go to the bank and they tell me right there to download the application and let's open it right now. And right there they tell me when it appears, if you are in another place and you need to use a nearby bank and you open your application and this site appears and wants to use your, what is your location to give you access to nearby banks, I feel safe because there is someone else who is telling me.
- Y cuando me siento insegura es cuando te digo, me aparece la aplicación, pero no la, cómo se llama, no la conozco. Solamente es como el anuncio y veo que me empieza a pedir como información mía y que quiere mi ubicación. Por ejemplo el del vaso, ¿no? Si el del vaso solamente me está diciendo solamente te vamos a enseñar a administrar tu agua, entonces, yo digo ¿para qué quiere mi aplicación y para qué quiere mi información? Entonces, ahí sí me sentiría incómoda, ¿no?
  - And when I feel insecure is when I tell you, I get the app, but I don't get the, what's it called, I don't know it. It's just like the ad and I see that it starts asking me for my information and it wants my location. For example, the one with the water glass, right? If the one with the water glass is only telling me that we are only going to teach you how to manage your water, then I say, why does he want my application and why does he want my information? Then, I would feel uncomfortable, wouldn't I?
- Bueno, yo pensaría que, ¿para qué quiere saber mi edad?
  - Well, I would think, why does it [the chatbot] want to know my age?
- Las respuestas ya están programadas y solo le responde. No hay nadie mirando su respuesta. ‘Pues, ahí sí no, ahí sí me la puso difícil porque si es algo de fiar, pues, estaría bien, pero si no, ahí sí ya me da miedo.’
  - The answers are already programmed and it only responds to you. There is no one looking at the answer. ‘Well, no, that really made it difficult for me because if it is something reliable, well, it would be fine, but if not, that really scares me.’
- Sí. Digo, porque a veces uno, pongamos que le tome una foto a mi marido y la vea primero la máquina.
  - Yes. I mean, because sometimes you, let's say I take a picture of my husband and the machine sees it first.
- Pues, yo, pues, así como te digo. Yo diría que está bien, pero por otra parte estaría mal porque sentiría que saben dónde estoy y dónde voy y todo. Me sentiría como vigilada.
  - Well, I, well, just as I say. I would say it's fine, but on the other hand it would be bad because I would feel that they know where I am and where I go and everything. I would feel like I was being watched.
- ¿Pero esa máquina no la mira la gente? ¿Como una persona no la revisa o así?
  - But don't people look at that machine? Like a person doesn't check it or something?
- Me sentiría como vigilada por alguien. Aunque dices tú está reprogramada y todo, pero en mí estaría la duda de que, ¿qué tal que si ese que está reprogramando sabe dónde estoy y anda aquí donde yo estoy?
  - I would feel like I was being watched by someone. Although you say it's programmed and all, but in me there would be the doubt that, what if that person who is reprogramming knows where I am and is here where I am?
- Pues, cosas personales como cuántos hijos tengo, cómo se llaman. Información de mis hijos o información mía, sí, eso ya no.
  - Well, personal things like how many children I have, what their names are; information about my children or information about me, that I would no longer share

**Physical activity barriers**

***English-speaking participants:***

- Because first off, if you have children, like I do, I have two children, and I can't leave this house until they're done with their schooling...And plus, you have to be in a place where there's Wi-Fi. So in order for you to workout and go to the park, you can't have Wi-Fi at the same time. So yeah, so it's really difficult. It makes it really difficult sometimes.
- I don't have to spend time on the street like going to schools or to work because I'm always here at
- home and I'm always busy also. But instead of like spend 20 minutes going to the school, those 20 minutes, I can just do something like work out here.
- That's hard for me because I have to wear a mask, and I don't like to wear a mask when I'm outside walking.
- I don't know how to use a bicycle. But [my children] use a bicycle and I have to run behind them with my mask. And that's something that I don't really like. So for me, that's like a challenge right now. And because I have concerns about safety right now.
- The normal life that we had before [COVID-19], I went out, I went to the store. I went to just going to any store to walk. It was really helpful. Now, I can't do that. My kids are always with me 24/7. And I don't take them to stores if I don't have to. I go to grocery store to buy groceries for the house, but that changed a lot. I cannot have the school hours to just go out and walk and do things, and move around. Like now, I go out once a week, probably. I do a lot of things at the house. I clean, and I clean outside. I do all kinds of things, but it's not the same. Just going out, getting out, getting down and out the car, walking through the aisles, and more physical things.
- I don't have the equipment to, perhaps, do something else here at the house.
- Because I live in LA County, I cannot go to the gym. And even when they were allowing us to, I said we weren't going because it was far too dangerous. So we're not going to the gym.
- We may have to replace our outdoor walking with indoor gym time as it gets--well, not indoor gym time--indoor exercise as the weather gets colder. I'm not terribly looking forward to that, but yeah. I don't think anyone is.
- Normally, it would be gym time, but we cannot afford to do that right now. So I'm walking two hours a day.
- It's not motivating to start exercising by myself.
- This month I haven't been doing as much, because I've been a little bit busier with work.
- Because of COVID, I canceled my [gym] membership in March--no, April. And I haven't renewed it because of COVID. I'm afraid to go because it's almost--yeah, I'm trying to be careful.

***Spanish-speaking participants:***

- Yo sufro mucho de la parte de la espalda baja y me gusta ver ejercicios que me ayudan en ese tipo de-- en la yoga
  - I suffer a lot from lower back pain and I like to see exercises that help me in that kind of...in yoga.
- ...yo no había de sentir ganas de hacer Zumba también porque no hay quien me motive de quién me espera para hacer Zumba o con quién voy a hacer o así.Y eso también ella dice está muy bien, ¿y sigue corriendo? Le digo, sí. De hecho, compré una corredora, le digo, porque en tiempos de frío no da ganas de hacer ejercicio.
  - ...I wouldn't feel like doing Zumba too because there's no one to motivate me as to who's waiting for me to do Zumba or who I'm going to do Zumba with or so...And that too she says is very good, and she keeps running? I tell her, yes. In fact, I bought a runner, I tell her, because in cold weather I don't feel like exercising.
- Sí, porque muchas veces hay personas que están bien deprimidas y los familiares no se arriman a ella porque no pueden ir a su casa o cosas así. Y enviarle un mensaje, que alguien le mandó un mensaje y ellos me imagino que se han de motivar. Decir, guau, alguien se acordó de mí.
  - Yes, because many times there are people who are very depressed and their relatives don't go to them because they can't go to their house or things like that. And sending them a message, that someone sent them a message, I imagine they must be motivated. To say, wow, somebody remembered me.
- Entonces, tengo que escribirlo, ¿verdad? No sé muy bien escribir, ¿verdad? Es que yo no estudié, no tuve mucha escuela.
  - So, I have to write it down, right? I don't know how to write very well, do I? I didn't study, I didn't have much schooling.
- Entonces, ya cuando empezó la pandemia fue muy estresante para mí porque tuve que mantenerme en casa, siendo que yo no estoy acostumbrada a estar todos los días en casa. Todos los días estaba fuera. Y al estar en la casa, pues fue muy difícil porque dejé de hacer las cosas. Tuve ansiedad. Sentí también depresión.
  - So, when the pandemic started, it was very stressful for me because I had to stay at home, since I am not used to being at home every day. I was out every day. And being at home, it was very difficult because I stopped doing things. I had anxiety. I also felt depressed.
- Y a pesar de que me siento como muy limitada, ¿no? De la cocina a la recámara, de la recámara al baño, del baño a la sala y solamente salir a lo necesario, si no hay necesidad de salir a otra cosa.
  - And even though I feel very limited, don't I? From the kitchen to the bedroom, from the bedroom to the bathroom, from the bathroom to the living room and only go out to what is necessary, if there is no need to go out to something else.
- Como no podemos ir a la escuela-- Antes íbamos a la escuela y hacíamos en la escuela, pero como hoy no podemos ir a la escuela por lo que estamos viviendo del coronavirus, ahora hacemos ejercicio, pero en casa por Zoom.
  - Since we can't go to school-- We used to go to school and do [exercise] at school, but since we can't go to school today because of what we are experiencing with the coronavirus, now we exercise, but at home through Zoom.
- Pues, es diferente. ¿Cómo le diría? Cuando no había nada de todo esto, pues, era diferente porque, pongamos, cuando llevaba a los niños a la escuela, pues, caminaba llevarlos, caminaba traerlos. Entonces, siempre andaba para allá y para acá. Y mis citas. Llevaba a mis 2 niños más grandes a la escuela a un lado y después iba a otro lugar con la otra niña más pequeña. Entonces, siempre andaba para allá y para allá. Caminaba mucho.
  - Well, it is different. How should I put it? When there was none of this [quarantine due to pandemic], well, it was different because, let's say, when I took the children to school, well, I walked to take them, I walked to bring them. So, I was always walking here and there. And walked to my appointments. I would take my 2 older children to school on one side and then I would go to another place with the other younger child. So, I was always going back and forth. I walked a lot.
- Y, entonces, ya cuando pasó todo esto, pues, ya todos fuimos al encierro y, entonces, ella extrañaba mucho eso y siempre se ponía triste, pues, porque yo ya no la llevaba a su escuela.
  - And, then, when all this happened, well, we all went to the enclosure [quarantine] and, then, she missed that a lot and she always got sad, well, because I didn't take her to her school anymore.
- Entonces, como le digo, sí nos afectó porque cuando tenía la oportunidad, íbamos a los parques, estábamos en los parques. Y, pues, ya ahora, pues, ya no.
  - So, as I say, it did affect us [pandemic] because when we had the opportunity, we went to the parks, we were in the parks. And, well, now, well, not anymore.
- Ha afectado bastante en el peso también porque hasta del peso nos hemos subido. No solo yo, sino que hasta los niños han subido de peso también. Entonces, sí nos ha afectado bastante.
  - It has affected our weight too, because we have even gained weight. Not only me, but even the children have gained weight too. So, yes, it has affected us a lot.
- para los niños, como le digo, también sería algo para los niños porque, pongamos, yo a ellos les digo aquí, vamos a hacer ejercicio y no quieren. Como que el cuerpo se le ha puesto pesado. Como que tampoco quieren andar haciendo ejercicio. Quieren estar puro sentados, acostados. No quieren andar activos. La que a veces anda un poco activa es la niña, pero antes como que hacía más actividad de hacer ejercicio y ahorita como también ya no mucho quiere hacer ejercicio. Tal vez porque el cuerpo se le ha puesto más pesado, no sé.
  - For the children, as I said, it would also be something for the children because, let's say, I tell them here, we are going to exercise and they don't want to. It is as if their bodies have become heavy. They don't want to exercise either. They want to just sit and lie down. They don't want to be active. The one who sometimes is a little active is the girl, but before she used to be more active and now she doesn't want to exercise much anymore. Maybe because her body has become heavier, I don't know.
- Pues, pienso que no hay una motivación porque no hay quien te motive. Si estás en la casa, solamente está. Y como ahorita que está también el frío, te pones a ver tele, te cobijas, te sientas en tu sillón. Te levantas, comes o vas a hacer lo que vas a hacer y de todas maneras estás comiendo. Y en cambio, sin la pandemia, la gente sale que a caminar al parque. Y más que ahora con la pandemia uno no puede salir y antes la gente salía, caminaba o había muchas clases de salones de Zumba, se iba la Zumba. Y ahora todo eso está cerrado.
  - Well, I think there is no motivation because there is no one to motivate you. If you are at home, that's all there is. And now that it is also cold, you start watching TV, you take shelter, you sit on your couch. You get up, you eat or you go to do whatever you are going to do and you are eating anyway. And on the other hand, without the pandemic, people go for a walk in the park. And more than that now with the pandemic you can't go out and before people went out, walked or there were many Zumba classes, there was Zumba. And now all that is closed.

**Ease of use after testing our prototype**

***English-speaking participants:***

- This [chatbot]'s a little more frustrating. Just because if you send it the wrong message, or you respond yes or no. Like right now, when I said "none," it sent me back to "let's get started."
- The questions on this one are like really, really extensive...And this [chatbot] doesn't really understand when you put like a short answer. You have to put like specifically what they want you to answer.
- I like [the chatbot] because it's fast and at the moment.
- So I texted, and the question was how will you say that social support motivates you to exercise, and I put in “a lot.” And they sent me back, “Hi, I am your physical health companion.” So they sent me, again, the same message that they sent me at first.
- I'll type yes and see what happens. And then it started over. I'm sorry. It started completely over.
- I'll get through it. I broke the script on accident.
- I mean, it was quick responses, which is good. It can be a quick conversation because you're not waiting for somebody to type something out, right? It's not like you talking to me and us was texting back and forth and me waiting for you to type of response, which could take five minutes or whatever, and so it's quick, right? So I feel like it's a quick conversation and you get to set the pace, and it's just very responsive. So you don't have to wait, which is cool for people who like things super quick. So in that case, I feel like that's beneficial. So, yeah, I didn't feel like it was like a long conversation.
- I think the [chatbot] went a little faster because the [Wizard of Oz], to me, was slow. [The Wizard of Oz] just kind of like--when I typed in my answer, it was a little slow to me. The [chatbot] seemed to go a little faster… once I typed in my response, then it was quicker to get a response.

***Spanish-speaking participants:***

- Las preguntas estaban bien. Como, ¿en qué te puedo ayudar? Se hicieron bien.
  - The questions were good. Like, how can I help you? They were well done.
- ¿Cuál es su reacción o cómo ve que una máquina le estaba contestando a sus respuestas? Pues, no se siente nada diferente. Se siente bien, tal vez porque como uno ya se va adaptando a las cosas que están viniendo, entonces, yo pienso que todo está bien. Para mí está bien. Porque como le digo, pues, pongamos, a veces uno tiene una cita y no se acuerda, pero algo así, un recordatorio de eso, pues, estaría bien.
  - What is your reaction or how do you see that a machine was answering your answers? ‘Well, it doesn't feel any different. It feels good, maybe because as one is already adapting to the things that are coming, then, I think everything is fine. For me it is fine. Because as I say, well, let's say, sometimes you have an appointment and you don't remember, but something like that, a reminder of that, well, it would be good.’
- Él primero [Wizard of OZ] como que me daba este respuestas y preguntas muy cortas. Muy cortas y muy específico, ¿no? O sea, lo que te pregunto, me respondes. Y el segundo [el chatbot], el segundo sentí que me daba más motivación y como que interactuaba un poquito más con mi persona. OK. ¿Cuál diría usted que prefería si tenía que elegir una? El segundo [chatbot].
  - ‘The first one [Wizard of OZ] kind of gave me these very short answers and questions. Very short and very specific, right? I mean, what I ask you, you answer me. And the second one [chatbot], the second one I felt like gave me more motivation and kind of interacted a little bit more with myself. OK.’ Which one would you say you preferred if you had to choose one? ‘The second [chatbot].’
- Me sentí bien. Me sentí cómoda con la aplicación porque que es como si estuvieran platicando con alguien, pero no es alguien.
  - I felt good. I felt comfortable with the [chatbot] application because it's like you're chatting with someone, but it's not someone.
- Pues yo creo que me gustó mucho, pero, sí, te digo, el segundo [chatbot] programa me gustaría también-- Como abarca un poquito más de lo que haces y todo eso. Como que el segundo programa me gusta porque quiere meter tu ejercicio en una hora que sea práctico para ti.
  - Well, I think I liked it a lot, but, yeah, I tell you, the second [chatbot] program I would like too--like it covers a little bit more of what you do and all that. Like, I like the second program because it wants to put your exercise in an hour that is practical for you.
- ¿Y usted se envisionaría a lo mejor mandándole textos como afuera del estudio? ¿Como si en realidad esto se creara más fuerte y usted sí usaría esto? ¿O todavía se le hace muy nuevo? ¿O cómo lo ve? No, yo creo que sí lo usaría. Yo pienso que yo sí lo usaría. Ajá. Porque sí me pareció bien que logre esto.
  - And would you envision maybe sending texts like outside the study? Like if it actually developed more completely, would you use it? Or is it still too new to you? Or how do you see it? ‘No, I think I would use it. I think I would use it. Because it seemed good to me that we can achieve this [referring to the chatbot itself].’

**Satisfaction, usefulness, and humanness.**

***English speaking participants:***

- I think that's great to have someone to coach me and someone to be able to answer questions.
- It was more like a computer talking to you. Like really, really physically a robot talking to you that doesn't really understand what you're saying, doesn't get your answers. And if they do have like a way to answer fast, they don't get there. They don't understand their own answers, so I don't know. Kind of weird.
- I think it's asking for a lot of information instead of going to the point.
- [The chatbot] feels like it's just a computer who answers fast.
- [The chatbot] was going more around the question and not really giving me any solutions.
- They're very in-depth up until the break point.
- Yeah, [the chatbot] was trying to motivate so you could get more active. And it was giving you ideas of what to do [based on] what your response was. I said "walking" and it said, more ideas as hike, get together with friends, and all that stuff.
- I think this was a little bit better. The second one. Even though I had a little more problems, it was a little more specific or more detailed (than the Wizard of Oz).
- But thing I didn't like about the [Wizard-of-Oz] was that the response time wasn't as fast as the [chatbot]. And also, there was like grammatical errors in the [Wizard of Oz]'s responses. And there weren't any grammatical errors in the chatbot's responses.
- [The chatbot]'s pretty conversational, so that's nice.
- The [chatbot] that was kind of tech-y, it still had good information, and it was trying to get specific type of answers so that it could help. So it wasn't a problem with it.
- [The chatbot] asks a lot of questions, but if your goal is to really utilize this in a way that you're going to sit and it's as if you're talking to somebody, then I feel like that's how a normal consult would go if you're sitting with somebody. They ask all those questions anyway. I think it was fine. Like I said, it's quick, which I think is great. I think it's perfect, I guess.
- I think [this chatbot] would do better than just a fitness tracker.
- This is very innovative.
- I think it's too much--it's asking too many questions. It's just too much.
- I like that because then I have some--I'll have, like--it's like having a friend. And you rely on that person, you know? And you're my support person. But the chatbot would be my support person.

***Spanish-speaking participants:***

- Bueno, el [chatbot] segundo interactúa más con mi persona, pero se podría decir que el primero me respondía a la mejor un poquito más directamente a lo que yo preguntaba. O sea, por ejemplo, el primero luego luego me dio recomendaciones, me mandó luego luego el video, un enlace al video de YouTube, para que indagara directamente lo que yo a lo mejor le estaba preguntando
  - Well, the second [chatbot] interacts more with me, but I could say that the first one [The WoZ] answered me a little more directly to what I was asking. I mean, for example, the first one [WoZ] then gave me recommendations, then sent me the video, a link to the YouTube video, so that I could directly investigate what I was asking him.
- Y el otro[ el chatbot] me preguntaba un poquito más sobre cómo llegar al punto, al punto de lo que yo quería llegar, o sea, de decirme ¿sabes qué? Vas a hacer esto, vas a hacer lo otro, sí quería saber un poquito más de lo que yo hacía. Como que me gustaría que fuera como la combinación de que a la vez que quiere saber un poquito más de lo que yo hago, también me dijera OK, mientras estás lavando, a la mejor, puedes estar viendo este video de YouTube.
  - And the other [chatbot] was asking me a little bit more about how to get to the point, to the point of what I wanted to get to, that is, to tell me you know what? You are going to do this, you are going to do that, he wanted to know a little bit more about what I was doing. I'd like it to be like a combination of him wanting to know a little bit more about what I do, but also telling me OK, while you're washing, maybe you can watch this YouTube video.
- Perdón que te interrumpa, pero fíjate que eso que acabas de decir siento, de verdad, que sí es muy importante porque hay un aspecto por ejemplo de lo que acabas de decir de esos mensajes. Por el hecho de que tengas un recordatorio, como te decía yo, OK, si puedo mantenerlo.
  - Sorry to interrupt you, but notice that what you just said, I really feel that it is very important because there is an aspect, for example, of what you just said about those messages. For the fact that you have a reminder, as I was telling you, OK, I can maintain that [referring to maintaining an exercise routine and happy moods via text reminders].
- Pero cómo dices, ¿qué tal que ese día no tengo ánimos? ¿Qué tal ese día estoy bien apasionada y no quiero hacer absolutamente nada? Y recibo el recordatorio y necesito contestarle y le puedo decir oh, lo siento, hoy no me siento bien. Y sí, sí me gustaría que llegara un mensaje, de decir, no, todo está bien, tú puedes. No importa que estés así. O sea, un mensaje de aliento, fíjate que sí, sí estaría muy bien.
  - But like you say, how about that day I'm not in the mood? How about that day I'm really not passionate and I don't want to do absolutely nothing? And I get a reminder [from the chatbot] and I can say oh, I'm sorry, I'm not feeling well today. And yes, yes I would like to get a message, to say: ‘no, it's all right, you can do it. It doesn't matter that you are like this.’ In other words, a message of encouragement, yes, yes, that would be great.
- Pues, me siento bien porque ahora voy más tranquila de saber eso. Yo no lo entendía muy bien y ahora sí lo voy a entender bien.
  - Well, I feel good because now I feel calmer knowing that. I did not understand [the chatbot] very well and now I will understand it well.
- Estaría bien a la vez porque así como también quiero bajar de peso, estaría bien.
  - It would be nice at the same time because just as I also want to lose weight, it would be nice [on having a fully developed chatbot available].
- Mi respuesta, le respondí los años que tengo. ¿En letra o en número? El número. ¿Y no le ha contestado? No. Se espantó yo creo con los años.
  - ‘My answer, I responded [to the chatbot] how old I am.’ In letters or in numbers? ‘The number.’ And he didn't answer you? ‘No. I think he got scared with the years.’
- Yo pienso que eso sí me gustaría, o sea, el saber que es algo, aunque sea una máquina, un chatbot, como tú dices, que no podría investigar más allá de la información que tú le das, pues sí me sentiría un poquito más confiada. Y que me mande así como textos de alerta de decir no llegaste a tu meta, te falta, a veces. Pues sí, sí me gustaría. Sí me parecería bien como probarlo.
  - I think I would like that, that is, to know that it is something, even if it is a machine, a chatbot, as you say, that could not investigate beyond the information you give it, then I would feel a little more confident. And that it would send me alert texts to say you didn't reach your goal, you are missing, sometimes. Well, yes, yes I would like that. Yes, I would like to try it out.
- ¿Usted estaría de acuerdo que le ayudaría a usted esa información? Pues, sí, sí ayudaría.
  - ‘Would you agree that this information would help you? Well, yes, it would.’
- Pues, bien. Como me alienta a hacer lo que yo no hubiera pensado hacer. Me gustaría. Estaría bien
  - Well, good. As it [the chatbot] encourages me to do what I would not have thought of doing. I would like to. It would be nice.
- El compañero de salud y ese sí es un programa. So por eso cada vez que le mandaba un texto casi al instante le mandaba una respuesta porque ya está programado si le pones sí, no, te regresa luego, luego…”Oh. Y yo pensando que esta [chatbot] era la persona.”
  - The health partner [the chatbot] is a program. So every time you sent it a text it almost instantly would send you a response because it's already programmed; if you put yes, no, he sends you back [the response] right away... "Oh. And here I thought this [chatbot] was the person."

**Sustainability**

***English-speaking participants:***

- I think it would have to be as long as I'm exercising, or...if I set a goal for like a month or two months, I think it would be great to have at least reminders or stuff like that, during the whole month. Or during these two months. So it would be useful.
- I think I will use [the chatbot] every day.
- If [the chatbot] don't keep up with my goal and they keep asking the same things and not moving on to the place that I am after, probably, two months from now, that will make me stop using it.
- Honestly, if [the chatbot] did some of the things that I can imagine asking it to do, I would probably use it a lot.
- *How long would you use the chatbot to see a change in your level of physical activity?* Maybe for a month?... I use them for a month, month to month.
- You know, you might get up and do some exercises, and you're like, well, let me try to get an idea of maybe something else that I can do to add to this workout. And it might change day to day. So I think--and throughout the day too. So I think that I would use [the chatbot] more.
- I feel like [the chatbot] is like a resource for people and if they find it to be helpful, then I think it will continue its interests. It's not like an app--because apps are not helpful, right? They're just data trackers, so I feel like you're tracking your data and like I don't really know how much--nothing like this where you're talking to somebody to help you work through things and think through things. Apps aren't really going to do that, so I don't think that it will have--I don't think it will be like fitness trackers, if that makes sense. I don't think it's going to have its initial excitement and then die off. I mean, it might like everything else, but I feel like if somebody for example like goes to see a personal trainer all the time, that doesn't like lose off. It's somebody to hold you accountable and help you when you get stuck sort of thing. So again, I don't know how much capacity this [chatbot] is going to have to really be that kind of accountability or whatever you want to call it, but it's not going to lose its excitement, I think, as much as a fitness tracker would, which doesn't talk to you at all, it just tracks your data.
- I will use [the chatbot]. I really would because I use MyHealth, the one that I track my miles and whatnot, every day. I look at it every day. And if I could get a reminder--and I put my goal in, and I'll say, I want to walk--I want to at least have 10,000 steps in today--I mean, in a week or day or whatever, in three days or whatever. And if I got a companion--if that chatbot can help me set up something where I don't have to do it all in one day, it'll tell me, well, if you want to reach your goal in three days, this is what you need to do. And I can utilize the chat box to help me, that would be helpful for me.

***Spanish-speaking participants:***

- Pues, yo trato de tomar todavía mi zumba porque eso me mantiene como-- yo me siento como contenta y como que los problemas que hay no me afectan mucho en la forma-- Uno se mantiene como activo. Y cuando no es así, uno se pone como agitado, como con demasiada hambre, como ansiedad y todas esas cosas que dan a la gente. Esa es la razón que yo trato de manejarme más haciendo ejercicio.
  - Well, I try to still take my zumba because it keeps me like--I feel like I'm happy and like the problems that are there don't affect me too much in the way--you stay kind of active. And when it's not like that, you get kind of agitated, like too hungry, like anxious and all those things that people get. That's why I try to manage more by exercising.
- Hay muchas mamás que dicen no, que yo no puedo hacerlo porque estoy bien ocupada. Es mentira. Y eso, oiga, es mentira. Imagínese, yo a mi hija la llevo, yo a mi niña la llevo a las 8:30 de la mañana a la escuela y yo ya hago mi zumba antes de llevarla. Es mentira eso [referring to that other people like many parents don't have a set structure or someone to help them so they feel like they can't exercise or work on their well being.]
  - There are many moms who say no, I can't do it because I'm too busy. That's a lie. And that, listen, it is a lie. Imagine, I take my daughter to school at 8:30 in the morning and I already do my zumba before taking her. That's a lie [referring to that other people like many parents don't have a set structure or someone to help them so they feel like they can't exercise or work on their well-being]
- Entonces, me gustaría porque, pues casi, como quien dice, después de comer uno se sienta a ver el teléfono, a ver esto, entonces, sí me gustaría porque me haría como un recordatorio. Por ejemplo, si yo como todos los días a las-- Ponle que ceno a las 5 de la tarde y me dice-- Y yo quiero hacer el ejercicio por la tarde, entonces, tendría mi recordatorio. Entonces, ya me tendría como activa.
  - So, I would like it because, well, almost, as they say, after eating one sits down to look at the phone, to see this, so, yes, I would like it because it would be like a reminder for me. For example, if I eat every day at 5 o'clock in the afternoon, and I want to exercise in the afternoon, then I would have my reminder. So, It [the chatbot] would already have me active.
- Me voy a ir a caminar, aunque sea aquí 2 calles de mi casa, los 20 minutos que es mi recordatorio. O sea, es como que sí me ayudaría [chatbot] mucho a mejorar en el aspecto de usar mi tecnología también.
  - I'm going to walk, even if it's 2 blocks from my house, the 20 minutes that is my reminder. I mean, it would help me [the chatbot] a lot to improve in the aspect of using my technology as well.
- Pues, cuando llegan mensajes así, cuando alguien le manda un mensaje a alguien que está como bien analizado, cosas así, tal vez ellos van a mirar como la vida diferente. Como decir, oh, me están motivando, hay que ponerle ganas para salir adelante o cosas así.
  - Well, when messages like that arrive, when someone sends a message to someone who is like well analyzed, things like that, maybe they are going to look at life differently. Like saying, oh, they are motivating me, you have to put your heart into it to get ahead or things like that.
- Entonces, a veces llamo y, pues, la pura máquina a veces contesta y le va dando instrucciones a uno. Entonces, sí, pues, muy bien. Porque cuando podamos y uno llama a la noche y, pues, no hay un empleado para contestar las llamadas, pues, la máquina está a cualquier hora.
  - Then, sometimes I call and, well, the machine sometimes answers and gives instructions. So, yes, well, very good. Because when we can and you call at night and, well, there is no employee to answer the calls, well, the machine is there at any time.
- En algún punto, pues, uno necesita como algo importante en la noche que, como dijo usted, un humano no pueda estar en ese momento, pues, si la máquina le-- ¿Cómo le diría? Ayudarlo a uno, pues. En ese momento, en ese momento, pues, sí estaría bien. Porque a veces uno en cualquier momento necesita de algo. Y, pues, si una persona humana no puede estar en ese momento, pues, si la máquina lo puede dar a uno, pues, sí está bien.
  - At some point, well, one needs say something important at night that, as you said, a human cannot be there at that time, well, if the machine would-- how would you say? Help you, well. At that moment, at that moment, well, yes, it would be good. Because sometimes you need something at any given moment. And, well, if a human person cannot be there at that moment, well, if the machine can give it [help] to you, well, yes, it would be fine.
- ¿Usted cree que algún día esto puede llegar a ese punto de poder mandar mensajes muy positivos para alentar a la gente? Yo pienso que sí. Que sí pueden hacerlo y que tengan esa aplicación para que la gente la vea y sepa de ella también.
  - Do you think that someday this can get to the point of being able to send very positive messages to encourage people? ‘I think so. That they can do it and that they have that application for people to see it and know about it too.’

**Content and usability recommendations:**

***English-speaking participants:***

- Visual videos or information or research or new information. Like, more material.
- Probably one thing is achieving the goal that you want to achieve, but you can go farther. So making challenges, I guess, on the app for you to do instead of just sticking to the goal and that's it.
- If [the chatbot] knows that I'm reaching my goals or getting close to reaching my goals, I'd probably want it to tell me “good job.” That kind of feedback. That would be one of the things I'd want it to tell me. The other thing I'd want it to tell me is, if I actually told it I had reached my [goal weight], the point that I was looking for, I would want it to tell me how to keep it going.
- Maybe send a video with it? Or a link? That would be kind of nice.
- You know, food is very important. Right now, I'm over eating, exaggeratedly, because I'm stressed. So maybe like, maybe, I don't know, some tips about how to eat better and eat less without going hungry or on a diet.
- Maybe just reminding me to stay active and do maybe 15 minutes at a time.
- Or maybe if [the chatbots] have access to YouTube, it's free, like suggesting links for different types of indoor exercises that they could do on this day that they're stuck in my house.
- I think it would be cool from an accountability standpoint… Maybe like a weekly check-in or something like that, so like how often would you like to check-in about this goal or something? Like weekly, bi-weekly, monthly. You know what I mean? Like maybe be an option like that and then the chatbot could be like, is it OK if we send you a message weekly, and you're like, yes.
- Like a daily reminder. “Would you like a daily fitness tip?”, or I don't know. Something like that. But yeah, I think some people would probably like that to like subscribe to something like that and it's also like a reminder to, oh, yeah, I need to get out and do my exercise today or whatever it might be. So I think things like that are helpful, all the little like motivational things and accountability things are usually helpful for people, at least I think.
- It could be a drop down box somewhere. If we're talking about--if I'm chatting about my, I'll say, physical activities, OK? And say physical activity is the main thing I want, is the caption. But under physical exercise or whatever, it could be a dropdown box where I could click on, do I want to talk about walking?

***Spanish-speaking participants:***

- Bueno, depende en la situación que esté viviendo, depende cómo esté viviendo, yo le dejaría un mensaje de que le eche ganas, que todo va a estar bien. Depende de la situación que esté viviendo.
  - Well, it depends on the situation they are living in, it depends on how they live, I would leave them a message that they should try hard, that everything is going to be fine. It depends on the situation she is living in. [on what messages they would send people to encourage them via the chatbot]
- Sí, que sean como la mitad porque es muy largo [el mensaje]. Pongamos ahora que, como le digo, como está largo, entonces, a la hora que va uno y, entonces, dice continuar, entonces, le pucha uno. Entonces, ya como que se pierde uno. ¿Dónde está el mensaje? ¿A dónde iba leyendo?
  - Yes, about half of it, because it is very long [the message]. Let's say now that, as I said, it is long, then, at the time you go and, then, you say to continue, then, you get lost, and you get lost. Where is the message? Where was I reading?
- Sí, se me haría bien saber cuánto es lo que uno se mueve. Lo que uno hace, pues, cómo moverse. Pues, me gustaría saber más sobre cuánto camina al día. Pues, no de quemar grasa, ¿verdad? Porque uno no quema grasa, pero sí es lo físico que uno no está nomás sentado, sino camina y saber cuánto se camina. Estaría bien.
  - Yes, it would do me good to know how much one moves. What one does, then, how to move. Well, I'd like to know more about how much you walk a day. Well, not about burning fat, right? Because you don't burn fat, but it is the physical aspect that you are not just sitting, but you walk and know how much you walk. That would be good.
- O también me puede como si pasara por donde venden comida y fuera saludable también sería lo mismo? ¿También como dijera, este lugar es bueno para que tú te alimentes o algo así?
  - Or I can also like if I pass by where they sell food and it is healthy it would also be the same? Also like I said, this place is good for you to nourish yourself or something like that.
- No, pues, yo creo que le voy a pedir [al chatbot] una información sobre cómo comer más saludable porque también tengo una caminadora y me subo también como 20 minutos ahí a correr.
  - No, well, I think I'm going to ask him [the chatbot] for information on how to eat healthier because I also have a treadmill and I go on it for about 20 minutes to run.
- Ah, eso luego con la velocidad del segundo y con el conocimiento del primero, ¿usted diría que sería mejor, verdad? Sí. Sí sería mejor.
  - Ah, then with the speed of the second [chatbot] and the knowledge of the first, you would say that would be better, right? Yes, it would be better.
- Pues, como ellas meterse allí y preguntarle sobre cosas, sobre ejercicios o la alimentación o cosas así saludables para ellas.
  - Well, like them getting in there and asking them about things, about exercise or nutrition or things like that that are healthy for them.
- Y si él no me las puede decir, o sea, puede decir, OK, hay un enlace que puedes seguir y ahí puedes encontrar variedad de alimentación que puedes usar para tonificar tus músculos.
  - And if he can't tell me, I mean, he can say, OK, there's a link that you can follow and there you can find a variety of foods that you can use to tone your muscles.
- también me gustaría, OK, puedes seguir de este enlace y puedes encontrar infusiones de agua con frutas sin azúcar, o sea, con pura fruta, pero sin adherir a los azúcares o frutas con algunos tipos de hierbas que sirven para hidratarte como la menta que le pone uno al agua, o qué sé yo. O sea, que nos lleve a enlaces. Me gustaría que nos vaya más a enlaces porque lo que me gustaría.
  - I would also like to, OK, you can follow this link and you can find infusions of water with fruit without sugar, that is, with pure fruit, but without adhering to sugars or fruit with some types of herbs that serve to hydrate you like mint that you put in the water, or what do I know. In other words, to take us to links. I would like it [the chatbot] to take us more to links because what I would like.
- O sea, me puede guiar a una página donde yo vea una foto con muchas infusiones de agua que tiene mucha frutas adentro y que me puedo tomar antes de hacer ejercicio o así.
  - I mean, it can guide me to a page where I can see a picture with a lot of water infusions that have a lot of fruits inside and that I can drink before exercising or something like that.
- Me gustaría que tratara también como de llevarnos a-- A guiarnos, a cómo alimentarnos, buscar recetas cómo alimentarnos mejor, pero él también diciéndonos OK, en la tarde vas a hacer esto, vas a hacer lo otro. Algo que yo le pregunte. No sé si me expliqué.
  - I would like him [the chatbot] to try also like to take us to-- to guide us, how to feed us, look for recipes how to feed myself better, but him also telling us OK, in the afternoon you're going to do this, you're going to do that. Something that I asked him. I don't know if I made myself clear.
- Bueno, tal vez, si soy una persona mayor, él me puede decir palabras como de adulto. Si soy una menor de edad, puede decirme palabras de niño. Se me hace bien.
  - Well, maybe, if I am an older person, he [the chatbot] can say adult words to me. If I am a minor, he can say childish words to me. That would seem right to me.

# Co-Design Workshop: *Charla Comunitaria*

We conducted a co-design workshop in Spanish. We called the conversation “Charla Comunitaria,” –a community chat to create a space for participants to freely share their thoughts freely.
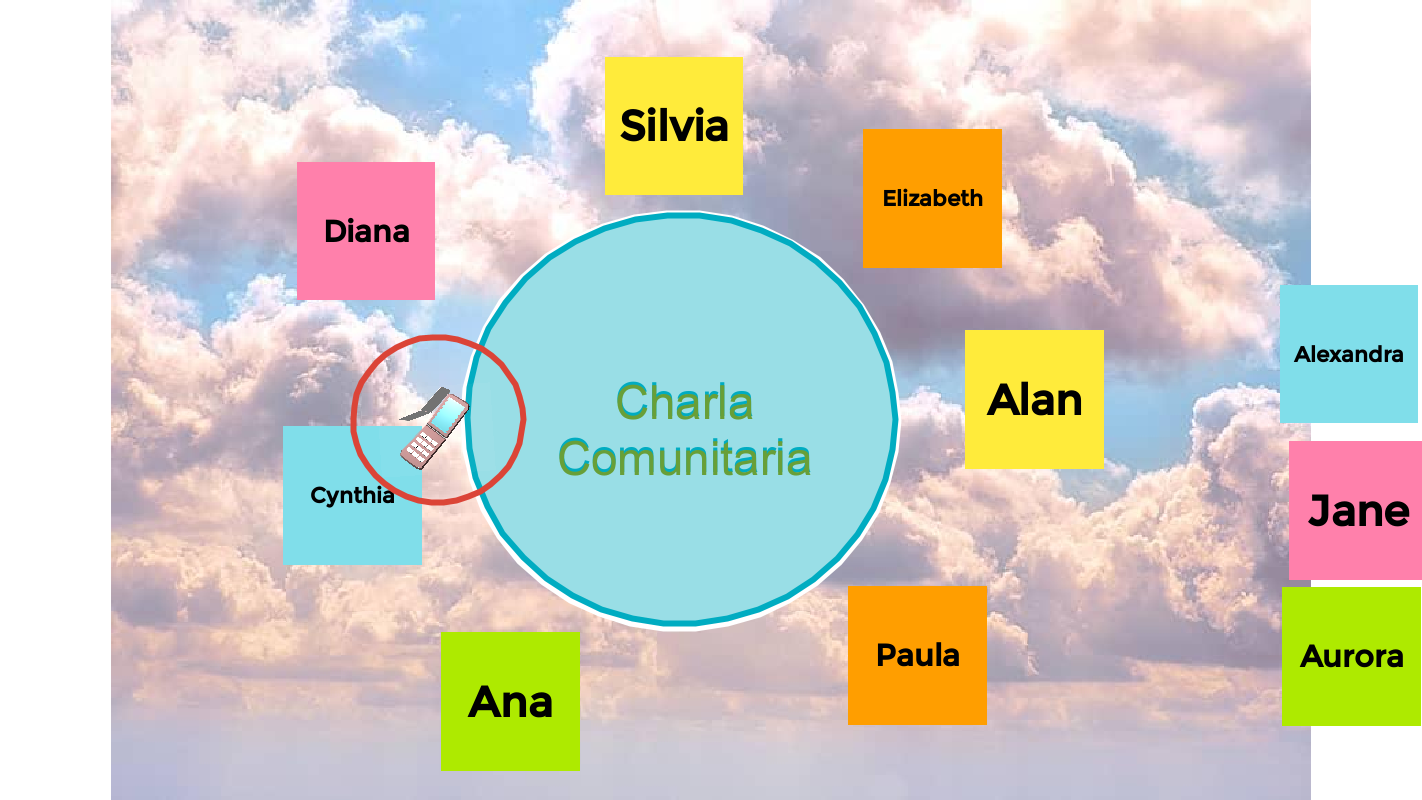


We created a virtual space to emulate and visualize the structure of an in-person co-design session. Each participant and researcher was represented by a sticky note, and a cellphone emoticon (circled in red) was used to show the group whose turn it was to speak. This image along with the Zoom conference was used to onboard and have participants interact/ introduce each other. Pseudonyms replaced real participants' names for privacy.

**Intro:**

A modified version of the Rose, Thorn, and Bud activity was used to introduce participants. Each participant introduced themselves, shared something good (rose), bad (thorn), and something they looked forward to(bud) regarding their mobile phones.

**
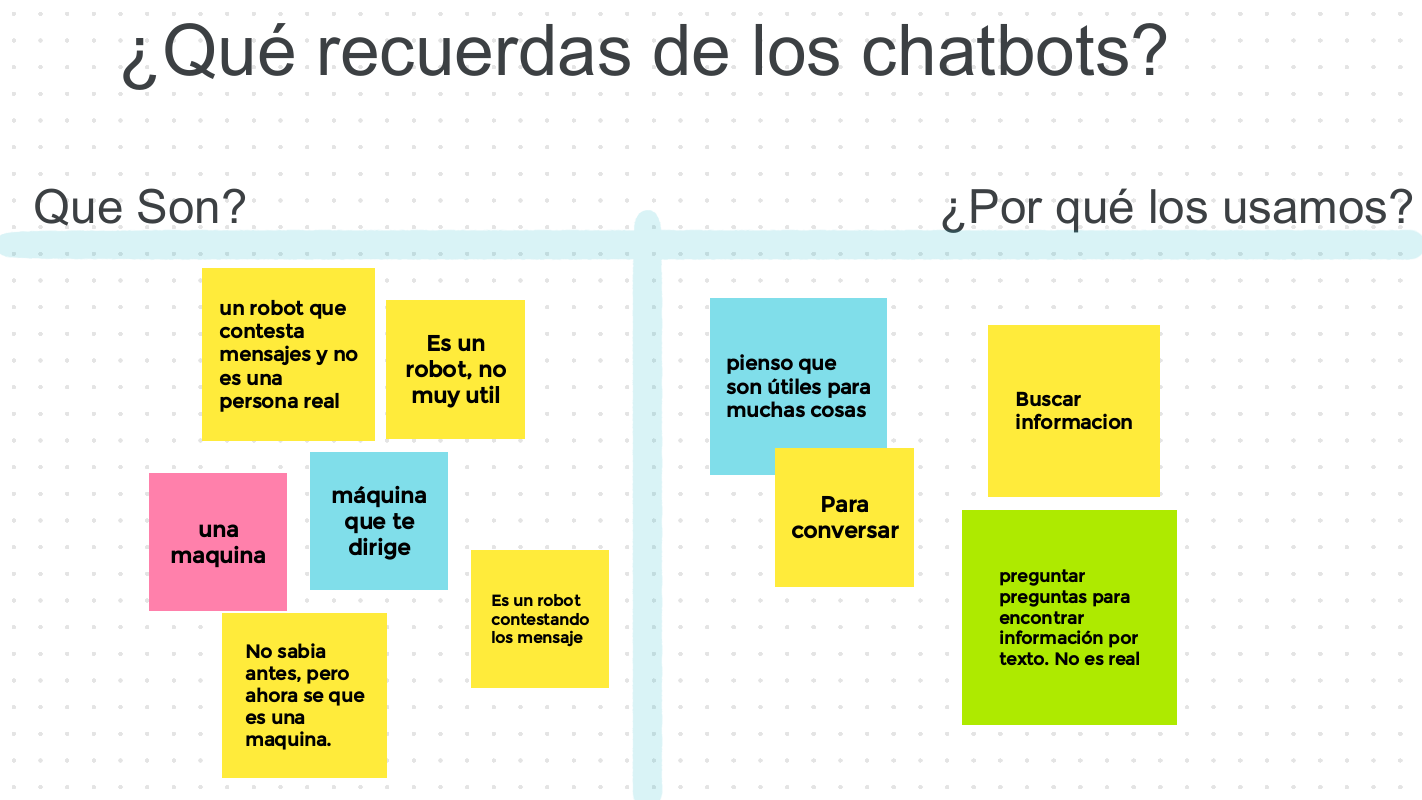
**

**Preference for conversational flows:**

Participants mentioned they would like the chatbot to help with a whole range of activities, including finding health information, cooking, finding COVID-19 vaccine information, anti-stress tips, remember payments, and connect them to a support group.

**
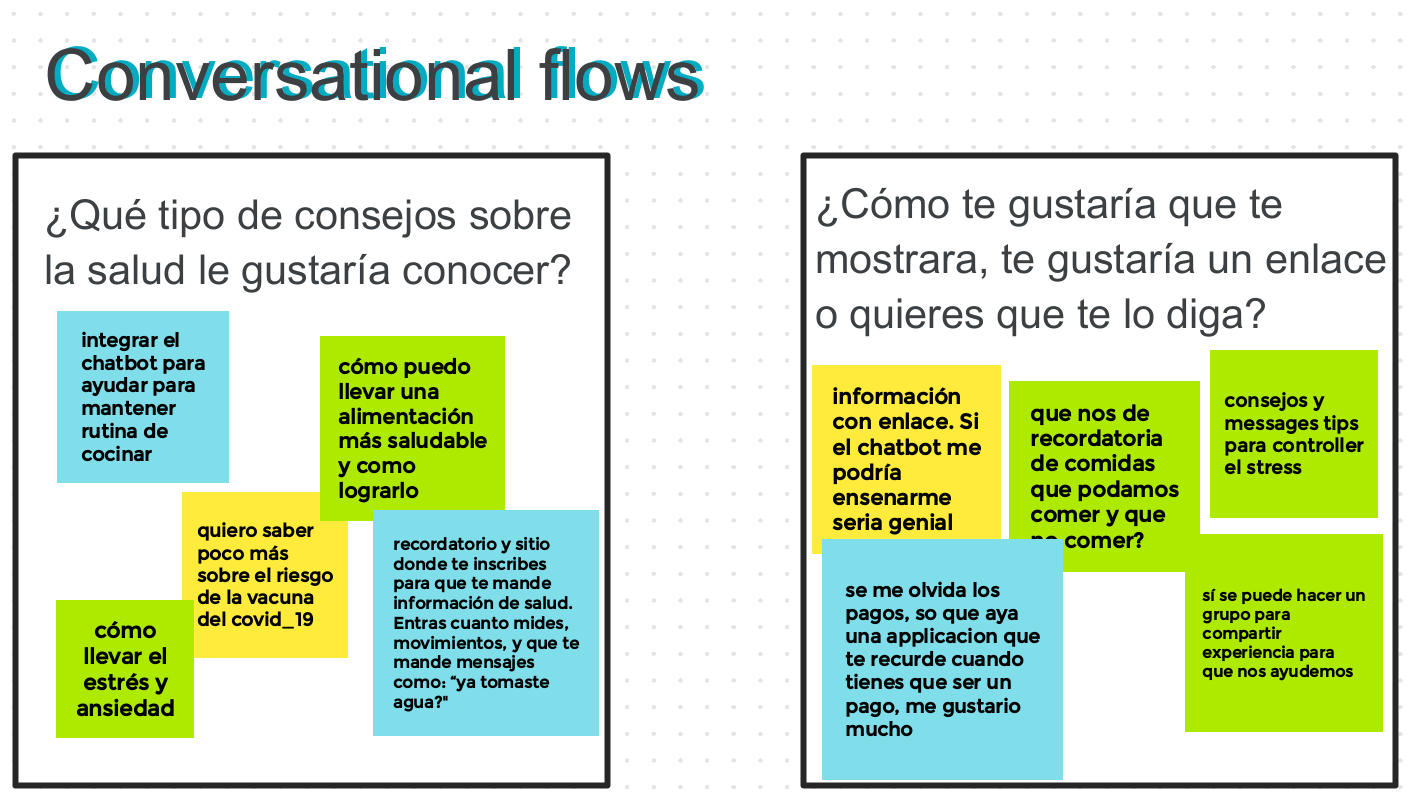
**

**What would you create?:**

**
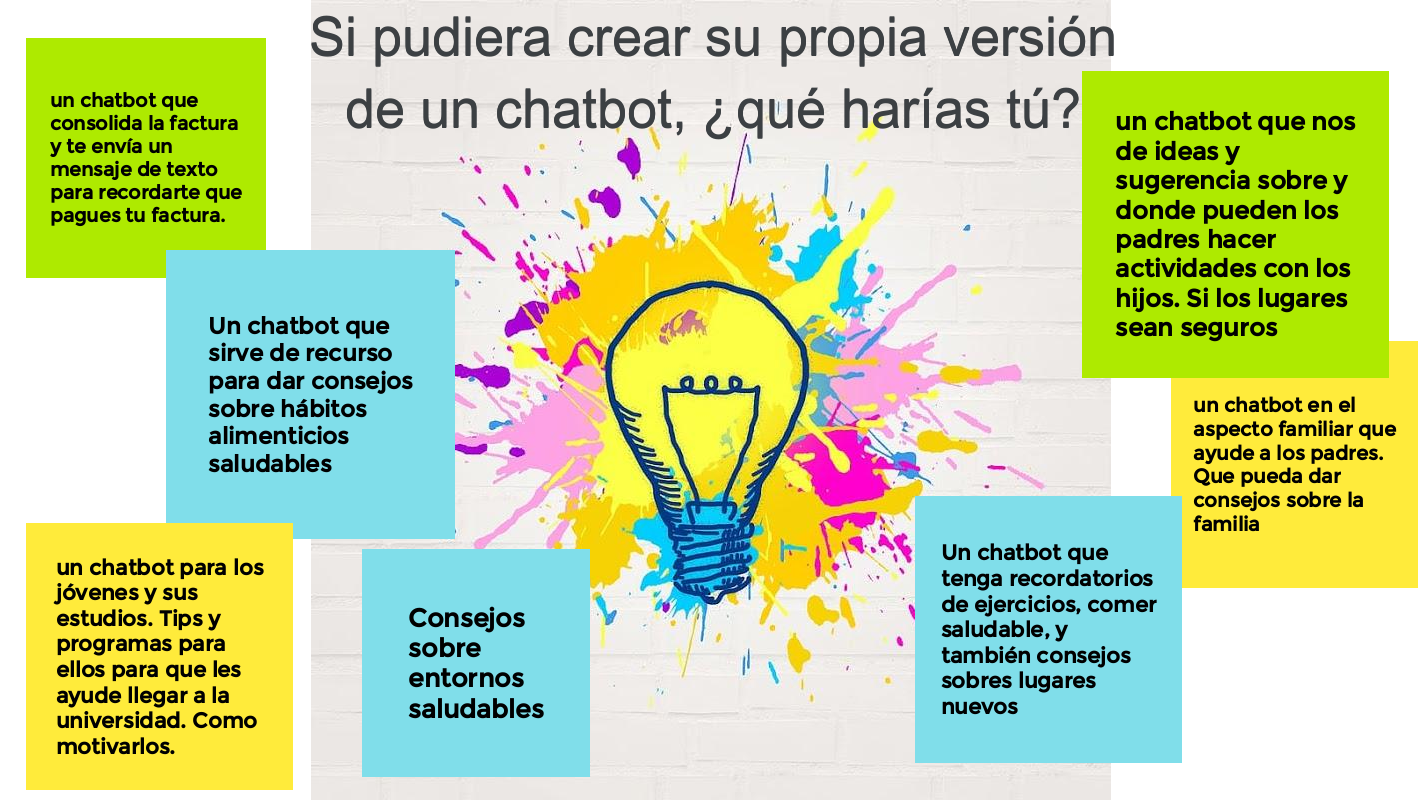
**

When asked what they would create participants gave a range of answers, including a chatbot that sends payment reminders, helps finding healthy food, gives advice about healthy environments, exercise and cooking tips, and recommendations for visiting new places. Finally, they would design chatbots that help young people study and go to university, and come up with activities for children and help with parenting.

##

## Translated Jamboard Session
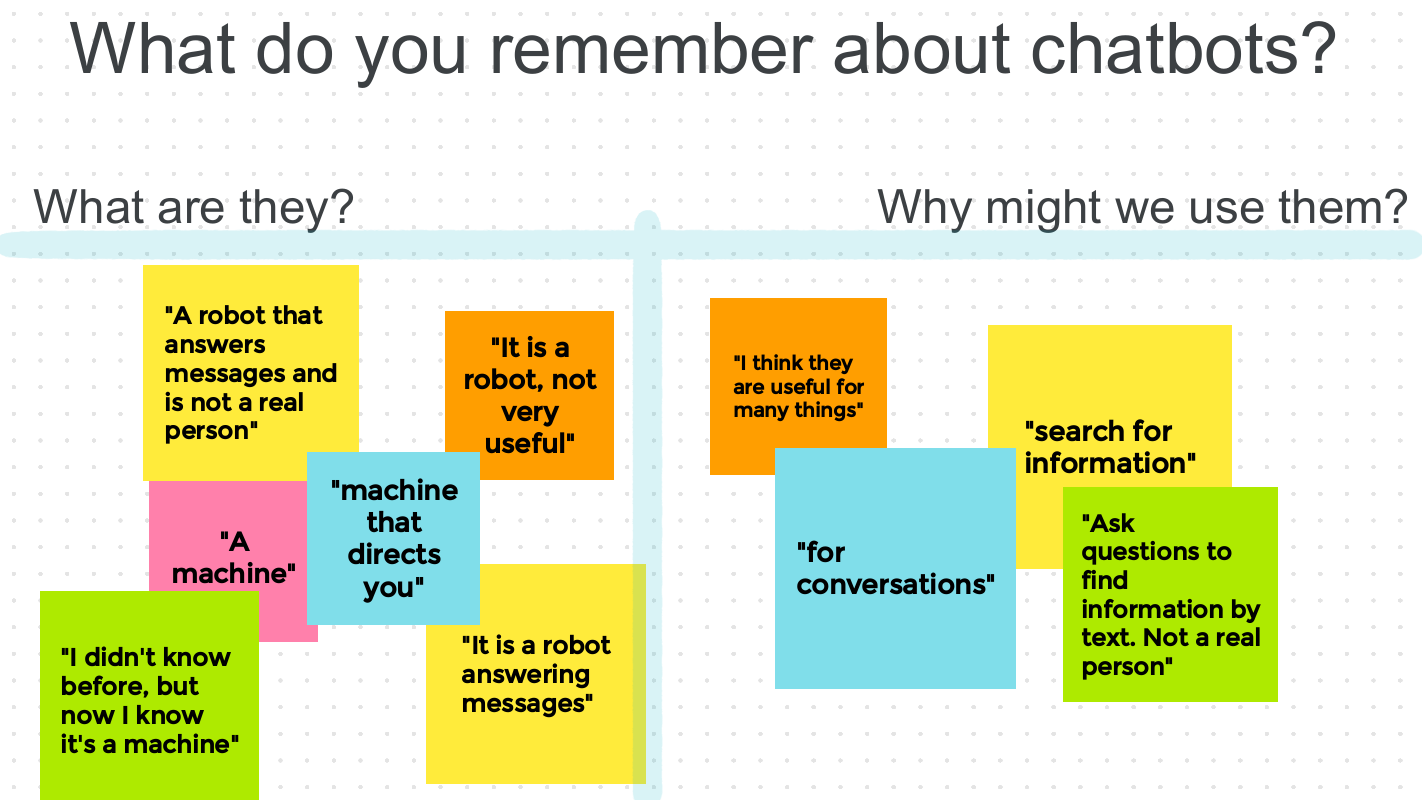


**
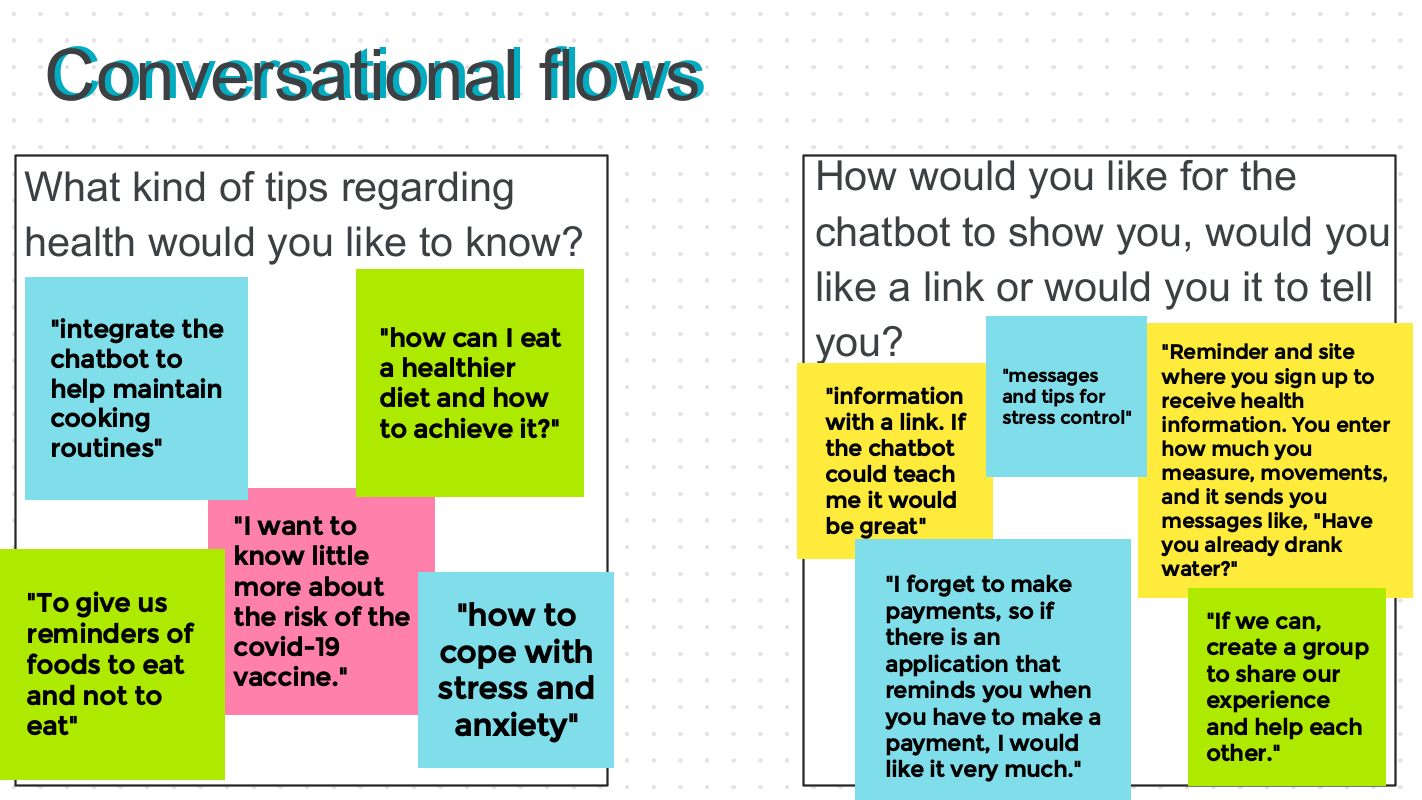

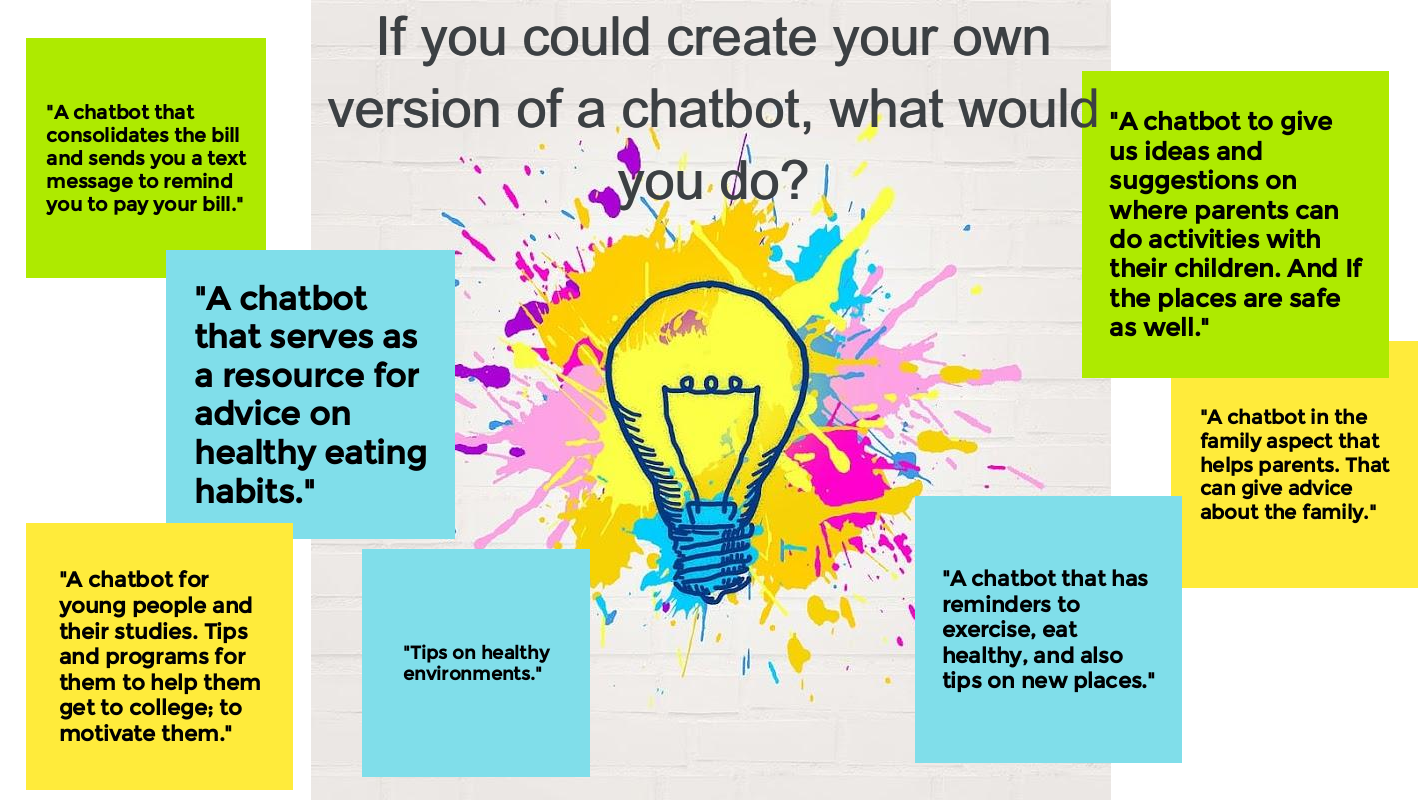
**
